# Multivalent display of minimal *Clostridium difficile* glycan epitopes mimics antigenic properties of larger glycans

**DOI:** 10.1038/ncomms11224

**Published:** 2016-04-19

**Authors:** Felix Broecker, Jonas Hanske, Christopher E. Martin, Ju Yuel Baek, Annette Wahlbrink, Felix Wojcik, Laura Hartmann, Christoph Rademacher, Chakkumkal Anish, Peter H. Seeberger

**Affiliations:** 1 Department of Biomolecular Systems, Max Planck Institute of Colloids and Interfaces, Potsdam-Golm Science Park, 14476 Potsdam, Germany; 2 Department of Chemistry and Biochemistry, Freie Universität Berlin, 14195 Berlin, Germany

## Abstract

Synthetic cell-surface glycans are promising vaccine candidates against *Clostridium difficile*. The complexity of large, highly antigenic and immunogenic glycans is a synthetic challenge. Less complex antigens providing similar immune responses are desirable for vaccine development. Based on molecular-level glycan–antibody interaction analyses, we here demonstrate that the *C. difficile* surface polysaccharide-I (PS-I) can be resembled by multivalent display of minimal disaccharide epitopes on a synthetic scaffold that does not participate in binding. We show that antibody avidity as a measure of antigenicity increases by about five orders of magnitude when disaccharides are compared with constructs containing five disaccharides. The synthetic, pentavalent vaccine candidate containing a peptide T-cell epitope elicits weak but highly specific antibody responses to larger PS-I glycans in mice. This study highlights the potential of multivalently displaying small oligosaccharides to achieve antigenicity characteristic of larger glycans. The approach may result in more cost-efficient carbohydrate vaccines with reduced synthetic effort.

## Abstract (web summary)

Immunologically-active glycans are promising vaccine candidates but can be difficult to synthesize. Here, the authors show that pentavalent display of a minimal disaccharde epitope on a chemical scaffold can mimic a native *C. difficile* glycan antigen, representing a simple approach to synthetic vaccine production.

## Graphical abstract

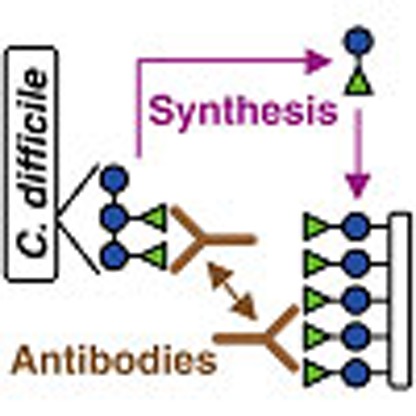

Immunologically active surface glycans expressed on bacterial, viral and parasitic pathogens are attractive vaccine targets^1^,^2^. Glycoconjugate vaccines consisting of isolated bacterial polysaccharides from *Streptococcus pneumoniae*, *Neisseria meningitidis* and *Haemophilus influenzae* linked to immunogenic carrier proteins save millions of lives each year^2^. However, isolated polysaccharides are heterogeneous, vary from batch to batch and can be obtained only for culturable pathogens^2^,^3^. Synthetic glycans provide an appealing alternative, as they are not limited to fermentable pathogens^3^,^4^,^5^,^6^ and allow for structure-based epitope design and refinement^7^,^8^,^9^,^10^. Still, the features of glycans that govern the production of protective and strong binding antibodies remain poorly understood^9^. Conventional glycan antigen design is a time-consuming trial-and-error process. Synthetic targets are selected based on biological repeating units of natural polysaccharides and immunologically evaluated in animal models^10^,^11^,^12^. If the resulting antibodies do not target the pathogen, different antigens are synthesized and tested. Vaccine antigens have to elicit antibodies of high affinity and/or avidity that are associated with disease protection^2^,^13^,^14^. Insights into the interactions of glycan antigens and antibodies are key for the rational design of synthetic carbohydrate vaccines^8^,^9^,^15^. Identifying minimal glycan epitopes, the smallest oligosaccharides recognized by antibodies, helps to reduce synthetic complexity *en route* to cost-efficient vaccines^7^,^16^. In recent times, a minimal tetrasaccharide epitope of the *N. meningitidis* serogroup W135 capsule was identified by chemical synthesis in conjunction with immunization studies^17^. The question whether multivalent display of minimal glycan epitopes of *C. difficile* may induce immune responses characteristic of larger glycans has not yet been answered.

We recently identified the minimal disaccharide α-L-Rha-(1→3)-β-D-Glc glycan epitope of the *C. difficile* polysaccharide-I (PS-I) surface polysaccharide, a promising vaccine target, by screening patient antibodies and murine immunization studies^7^. A vaccine against *C. difficile* is not yet available^18^ and limited expression of PS-I polysaccharide in bacterial cultures requires chemical synthesis to obtain glycan quantities sufficient for immunologic studies^7^,^19^,^20^,^21^,^22^. The synthetic repeating unit of PS-I, a branched pentasaccharide containing glucose and rhamnose^19^, is highly immunogenic, but its synthesis is laborious^7^,^21^,^22^. The disaccharide minimal epitope is easier to synthesize and can induce antibodies binding to larger PS-I structures, but is less immunogenic^7^. If linking of minimal disaccharides can mimic larger glycans, a new class of synthetic vaccine against *C. difficile* may result.

Here we show that disaccharides multivalently linked on a synthetic OAA scaffold^23^,^24^,^25^ are highly antigenic and induce antibodies to larger PS-I glycans in mice. Molecular-level insights into interactions of mono- and multivalent glycans with monoclonal antibodies (mAbs) were gained by combining glycan microarray, surface plasmon resonance (SPR), Interaction Map (IM), saturation transfer difference (STD)-NMR and isothermal titration calorimetry (ITC) experiments. The mAbs mainly interacted with the terminal rhamnose and the adjacent glucose of the disaccharide. In the pentasaccharide, two disaccharides are connected by a glycosidic bond. This linkage does not directly participate in antibody binding, but increases the affinity from micromolar (disaccharide) to nanomolar (pentasaccharide), probably due to an entropy-driven process. Pentavalent display of disaccharides on an OAA scaffold lead to enhanced affinity to mAbs compared with monovalent glycans mainly through avidity effects. The pentavalent OAA equipped with a peptide T-cell epitope of the CRM_197_ immunogenic carrier protein^26^ was able to induce antibodies in mice that recognized larger PS-I glycans.

Our findings provide experimental proof that artificially connecting minimal glycan epitopes can mimic larger glycan structures (Fig. 1). This is a crucial step towards simplified synthesis of rationally designed antigens for vaccines against *C. difficile* and other pathogens expressing repetitive polysaccharide antigens.

## Results

### Pentasaccharide 1 elicits mAbs in mice

**Figure 2:**
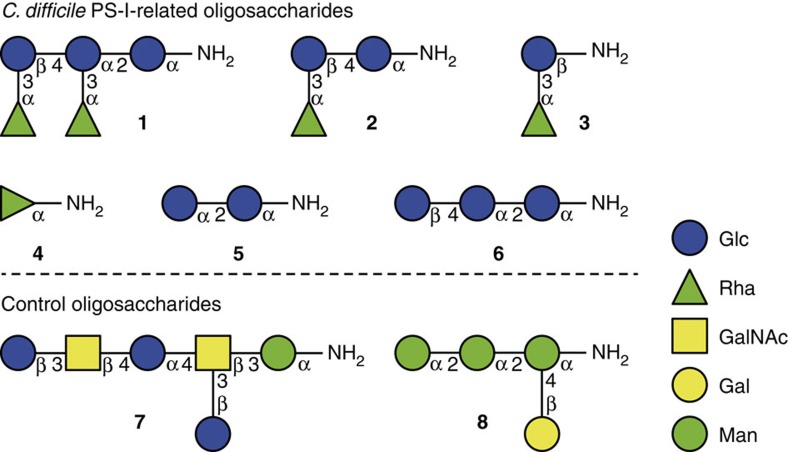
Oligosaccharides used in this study. The synthesis of *C. difficile* PS-I-related oligosaccharides **1**–**6** has been described previously^7^,^22^. *C. difficile* PS-II hexasaccharide^66^ **7** and the lipophosphoglycan capping tetrasaccharide from *Leishmania*^67^,^68^,^69^ **8** served as controls.

We recently described the syntheses of the *C. difficile* PS-I pentasaccharide repeating unit and oligosaccharide substructures^7^,^22^. The oligosaccharides were equipped with a reducing-end aminopentyl linker allowing for conjugation to carrier proteins and microarrays to study their immunologic properties (Fig. 2). Pentasaccharide **1** is the complete repeating unit of PS-I and disaccharide **3** is the minimal glycan epitope^7^.

**Figure 3:**
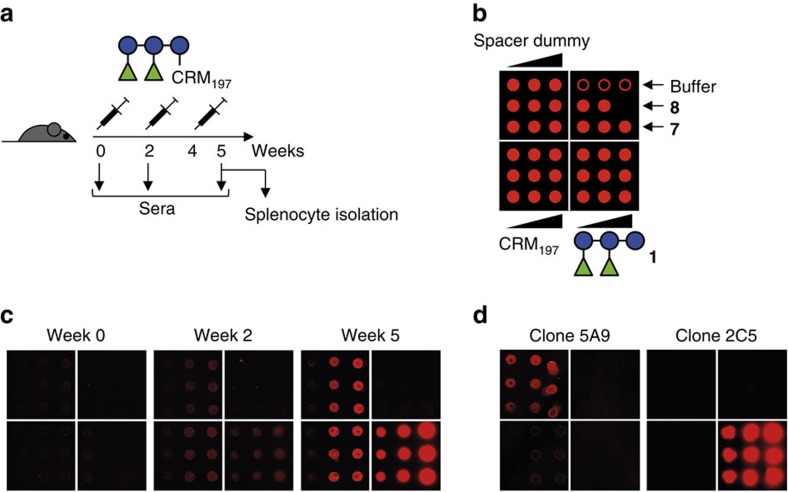
Generation of anti-PS-I mAbs. (**a**) Mice were immunized with pentasaccharide **1**-CRM_197_ glycoconjugate^7^ at indicated time points. Sera were collected for glycan microarray-assisted evaluation of the immune responses. Splenocytes of one mouse were subjected to hybridoma cell fusion^27^. (**b**) Spotting pattern of glycan microarrays presented in **c** and **d**. Buffer was 50 mM sodium phosphate, pH 8.5. Compound **1** was spotted at 0.1, 0.5 and 1 mM; CRM_197_ and the spacer dummy was spotted at 0.1, 0.5 and 1 μM; and **7** and **8** both at 1 mM. (**c**) Microarray scans representing the serum IgG response of one mouse. See Supplementary Fig. 1 for details. (**d**) Representative microarray scans of hybridoma cell supernatants of clone 5A9 producing antibodies to the spacer and 2C5 producing antibodies to PS-I.

To understand the structural determinants that mediate glycan–antibody interactions, we generated mAbs to PS-I with the hybridoma technique^27^ using a pentasaccharide **1**-CRM_197_ glycoconjugate^7^, to immunize mice (Fig. 3a). Custom microarrays presenting **1**, control oligosaccharides **7** and **8**, the carrier protein CRM_197_ and a dummy conjugate representing the immunogenic spacer moiety of the glycoconjugate^7^ (Fig. 3b) were used to follow serum IgG responses. The mice produced IgG antibodies to **1**, CRM_197_ and the spacer (Fig. 3c). Splenocytes of one selected mouse were used to obtain antibody-producing hybridomas^27^. Individual clones secreted IgG to either **1**, CRM_197_ or the spacer moiety without apparent cross-reaction (Fig. 3d).

**Figure 4:**
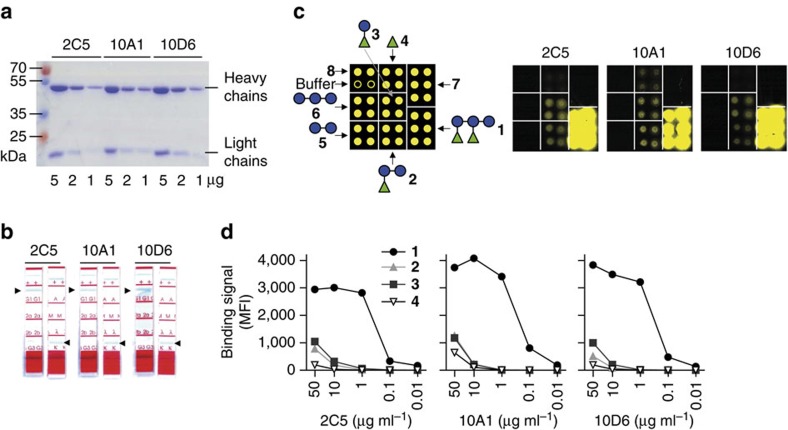
Characterization and epitope recognition patterns of anti-PS-I mAbs. (**a**) SDS–PAGE analysis of the purified mAbs showing bands corresponding to heavy and light IgG chains. (**b**) Isotyping analysis of the three mAbs. (**c**) Spotting pattern and representative microarray scans of purified mAbs at 10 μg ml^−1^. See Supplementary Fig. 2 for details. (**d**) Glycan microarray-inferred binding signals to selected PS-I antigens (**1**–**4**) expressed as background-subtracted mean fluorescence intensities (MFIs) are shown.

Three hybridoma clones termed 2C5, 10A1 and 10D6 produced IgG exclusively binding to **1**. SDS-polyacrylamide gel electrophoresis (SDS-PAGE) of the purified mAbs showed expected light and heavy IgG chains, and no apparent protein impurities (Fig. 4a). The three mAbs were of the IgG1 subtype (Fig. 4b). Microarrays with PS-I oligosaccharide substructures were used to determine their binding specificities (Fig. 4c). Highest binding signals to **1** and weaker signals to rhamnose-containing substructures **2** and **3** were observed. mAb 10A1 also weakly bound to mono-rhamnose **4**. No binding to oligoglucoses **5** and **6**, or unrelated glycans **7** or **8** was seen (Fig. 4c,d). Based on these findings, we concluded that the requirements for anti-PS-I antibody binding were a terminal rhamnose that is 1→3 linked to a glucose, as present in glycans **1–3**.

### mAbs bind to 1 with nanomolar affinity

**Figure 5:**
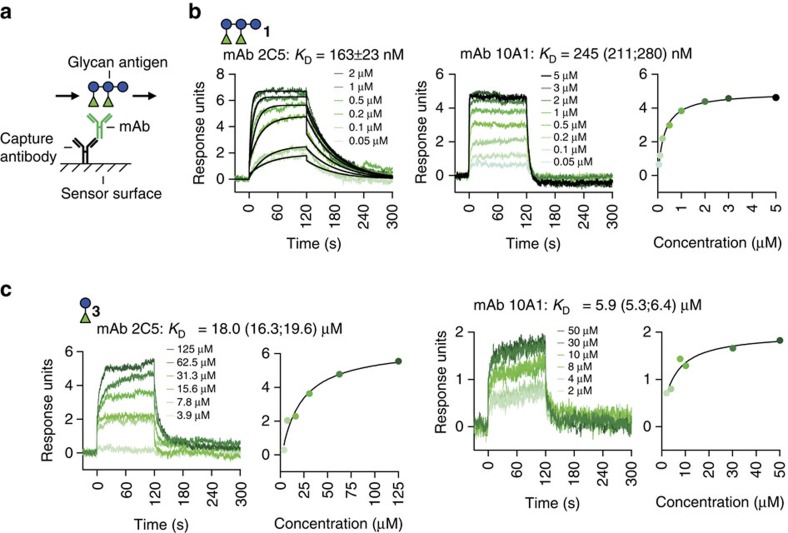
*K*_D_ values of anti-PS-I mAbs binding to 1 and 3 inferred by SPR. (**a**) mAbs were captured with an anti-mouse IgG antibody immobilized on the SPR sensor surface. Glycan antigens were passed over the surface to monitor changes in the response unit signals^8^. (**b**,**c**) Representative sensorgrams showing reference-subtracted binding signals to **1** (**b**) and **3** (**c**). *K*_D_ values were calculated by fitting the curves to a 1:1 Langmuir binding model (black overlaid curves) or with a steady-state affinity model (response units versus concentration plots). Indicated *K*_D_ values are mean±s.e.m. of *n*=3 or mean with minimum and maximum values in parentheses of *n*=2 independent experiments. See Supplementary Figs 3–5 for details.

Kinetic SPR experiments were performed to determine the binding strengths of the mAbs to PS-I glycans expressed as dissociation constants (*K*_D_)^8^ (Fig. 5a). *K*_D_ values to **1** were in the nanomolar (163–245 nM) (Fig. 5b) and those to **2** (13.3–18.8 μM) and **3** (5.9–37.5 μM) in the micromolar range (Fig. 5c). Weak or no binding was observed for mono-rhamnose **4** and oligoglucose **6**. Overall, the SPR measurements (summarized in Table 1) confirmed results obtained by glycan microarray presented above.

### mAbs bind via terminal Rha and adjacent Glc

**Figure 6:**
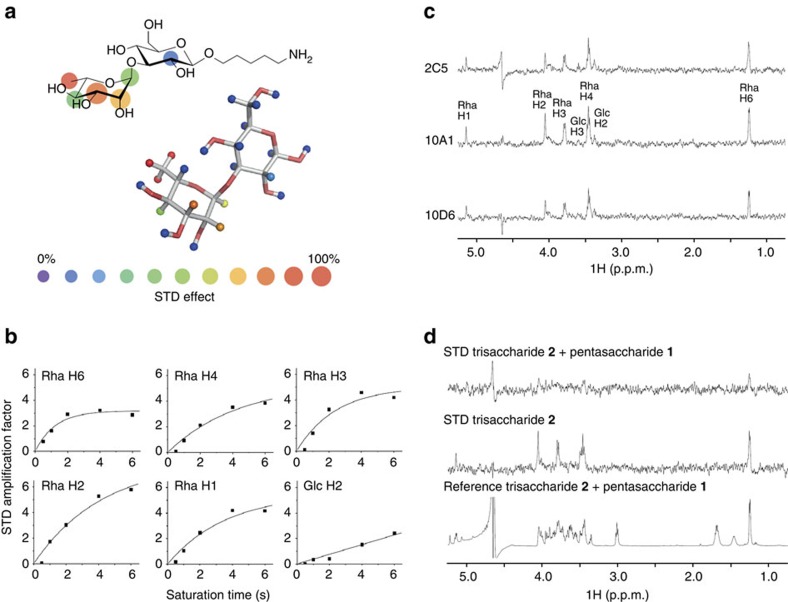
Epitope mapping of glycan–mAb interactions by STD-NMR. (**a**,**b**) Recognition of disaccharide **3** by mAb 10A1 shown as Lewis structure and three-dimensional model calculated with Glycam^70^. Colours indicate percentages of STD effects^28^. (**c**) Recognition patterns of **3** by three mAbs. STD spectra were acquired at 2 s saturation transfer time in the presence of mAbs 2C5, 10A1 and 10D6 (from top to bottom). (**d**) Competition of trisaccharide **2** by pentasaccharide **1**. Compound **2** exhibits STD effects on the same protons as **3** (middle panel). Addition of **1** resulted in the disappearance of the peaks imposed by **2**, indicating competition for the same binding site (upper panel). The lower panel shows the reference spectrum.

The glycan microarray and SPR data suggested the necessity of a terminal rhamnose and the adjacent glucose for antibody binding, whereas glucose moieties *per se* were immunologically inert. To understand antibody binding at molecular-level detail and to identify the functional groups involved in antibody binding, we performed STD-NMR measurements^28^. STD effects imposed by antibody binding to disaccharide **3** suggested that the main contact surface area was located within the terminal rhamnose, with highest STD effects at the indistinguishable methyl protons H6, H'6 and H''6 (100% normalized STD effect), as well as H3 (99%) and H2 (78%) (Fig. 6a,b). The glucose moiety in **3** contributed weakly to antibody binding around H3 (11%). Despite the low saturation transfer, this interaction appears to be important for antibody binding, as mono-rhamnose **4** was not or only weakly bound by the mAbs in glycan microarray and SPR experiments. Comparison of STD-NMR spectra with **3** at a fixed saturation time indicated that all mAbs recognized a similar epitope (Fig. 6c). In addition, trisaccharide **2**, which contains **3** and an additional glucose at the reducing end, showed comparable STD effects with mAb 10A1. This indicated that the additional glucose did not participate in binding and may explain similar binding affinities to **2** and **3** in SPR experiments described above.

Owing to the slow off-rates of **1** with all three mAbs, we were unable to determine any STD effects for the pentasaccharide. However, competition experiments of trisaccharide **2** with **1** completely diminished any STD effects imposed by **2** (Fig. 6d). This indicated identical binding sites and confirmed high-affinity binding of **1**. Overall, STD-NMR data confirmed antibody binding to **1**–**3** and showed that interactions were mainly mediated by terminal rhamnoses with weak but essential participation of adjacent glucoses. This suggested that strong binding to **1** was proably achieved by linking two disaccharide **3** subunits via a glycosidic bond that does not interact directly with the antibodies.

### mAb binding to 1 is entropically favoured

**Figure 7:**
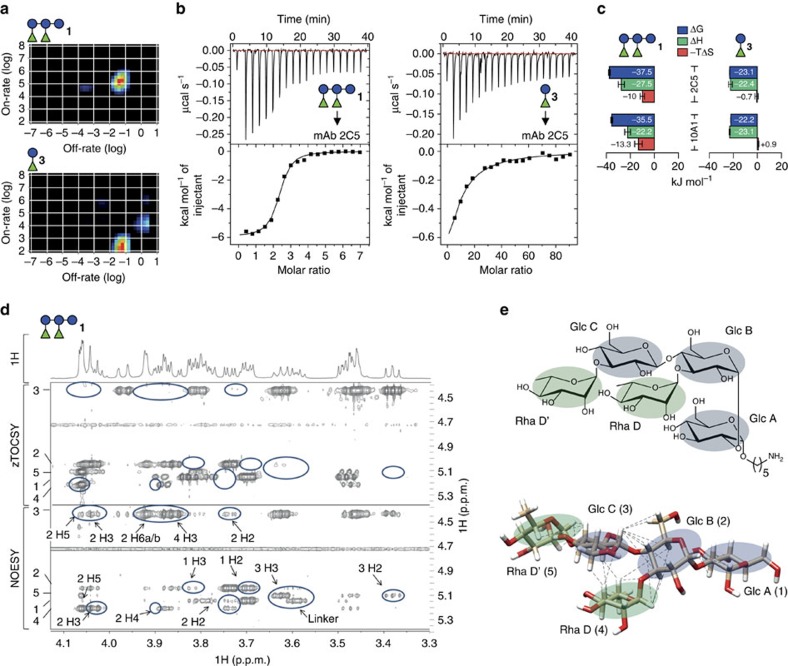
IM and thermodynamic analysis of glycan–mAb interactions and conformation of 1. (**a**) IM analysis. The heat maps represent on- and off-rates of mAb 2C5 to **1** (upper) and **3** (lower). (**b**) Thermograms acquired using 7 μM of mAb 2C5 and titrating 250 μM of **1** (left) or 3 mM of **3** (right) at 25 °C. Thermodynamic parameters were inferred by nonlinear least-square fits of the data points. (**c**) Summary of thermodynamic parameters for **1** and **3** interacting with mAbs 2C5 and 10A1. Bars represent mean±s.e.m. of two independent measurements. See Supplementary Fig. 6 for details. (**d**) One-dimensional proton NMR spectrum, and 2D zTOCSY and NOE spectroscopy (NOESY) NMR spectra of **1** showing the coupled protons to the anomeric protons of the five residues with numbering according to the structure shown in **e**. Blue circles mark cross-peaks that appear in the NOESY spectrum, whereas not showing a corresponding peak in the zTOCSY. These peaks were marked as inter-residue NOEs and labelled by residue number and proton name. (**e**) Chemical structure (upper) and three-dimensional model of **1** obtained by using GLYCAM^31^,^63^ (lower). Glucose and rhamnose residues are highlighted blue and green, respectively. Dotted lines represent 23 inter-residue NOEs derived from a comparison of NOESY and zTOCSY spectra. All collected NOEs are within the 5-Å distance limit in the model structure (see Supplementary Table 1 for details).

Pentasaccharide **1** was bound with about 100-fold lower *K*_D_ values than the minimal glycan epitope **3**. This stronger binding may have been due to avidity (re-binding) effects, as **3** is contained twice in **1**, or by higher affinity. To address this question, we compared the binding properties by IM analysis based on SPR traces^29^. This data indicated that stronger antibody binding to **1** was mainly due to a higher on-rate of 126,000 M^−1^ s^−1^ compared with 250 M^−1^ s^−1^ for **3**, whereas both off-rates were in the same range (Fig. 7a). Both antigens interacted with mAb 2C5 in a 1:1-like manner, indicated by one dominant peak in the IM plots. Therefore, stronger antibody binding to **1** was due to increased affinity, not avidity, which would be characterized by differences in off-rates and additional peaks^29^. This suggested that the binding pockets of anti-PS-I mAbs accommodated the entire pentasaccharide **1** and not just disaccharide **3**, which could explain higher affinity to **1**.

Assuming that **1** had more interactions with the binding pockets, association with **1** would liberate more water molecules during complex formation than **3**, thereby producing a more favourable entropy^30^. To investigate this, thermodynamic parameters of mAb interactions with **1** and **3** were measured by ITC (Fig. 7b). The binding stoichiometries for **1** were about two for mAbs 2C5 (2.2) and 10A1 (2.6), as expected for antibodies with two similar antigen-binding sites. For **3**, the binding stoichiometry was set constant to 2, to fit the data points. Both **1**–antibody and **3**–antibody interactions were mainly enthalpically driven (Fig. 7c). Entropic contributions were favourable for **1**, but considerably lower or slightly unfavourable for **3**. The favourable entropic term of the **1**–mAb 2C5 interaction was confirmed by SPR analysis that yielded similar thermodynamic parameters as ITC (Supplementary Fig. 6b,c). Therefore, entropically favoured binding was probably responsible for the increased affinity to **1**, possibly because it provides more hydrophobic interactions through methyl groups of rhamnoses. The favourable entropic terms supported the notion that **1** fills the binding pocket of the mAbs.

IM analysis indicated that binding stoichiometries for **1** and **3** were identical, despite the fact that **1** contains two copies of **3**. Therefore, the mAb binding pockets probably do not provide two identical binding sites for **3**, but one binding site for **1** that may adopt a more complex conformation. To obtain structural insight into the conformation of **1**, two-dimensional (2D) NMR spectroscopy was employed (Fig. 7d). Inter-residue nuclear Overhauser effects (NOEs) were all in agreement with a model of the solution structure of **1** based on calculations using the GLYCAM06 force field^31^ (Fig. 7e). These data suggested that **1** adopts a conformation in which the two units of **3** are oriented in an angled position relative to each other. Taken together, IM, ITC and 2D NMR experiments indicated that the mAb binding pockets accommodate **1**, whereas **3** made fewer contacts, resulting in lower binding affinity.

### Compound 11 shows high-avidity mAb binding

The results indicated that high-affinity antibody binding to PS-I was achieved by covalently linking two disaccharides **3** that together adopt a specific conformation fitting into the mAb binding pockets. As the connecting glycosidic bond is not directly involved in antibody binding, this linker may be replaced with a synthetic scaffold to mimic larger PS-I structures. To investigate this, a synthetic oligomer displaying five disaccharide units was synthesized using a derivative of **3** with a reducing end thioethyl linker **9** (see Supplementary Note 1 and Supplementary Fig. 8 for the synthesis). Two disaccharide-functionalized OAAs^25^ were prepared: monovalent **10** and pentavalent **11** (Supplementary Fig. 7a).

**Figure 8:**
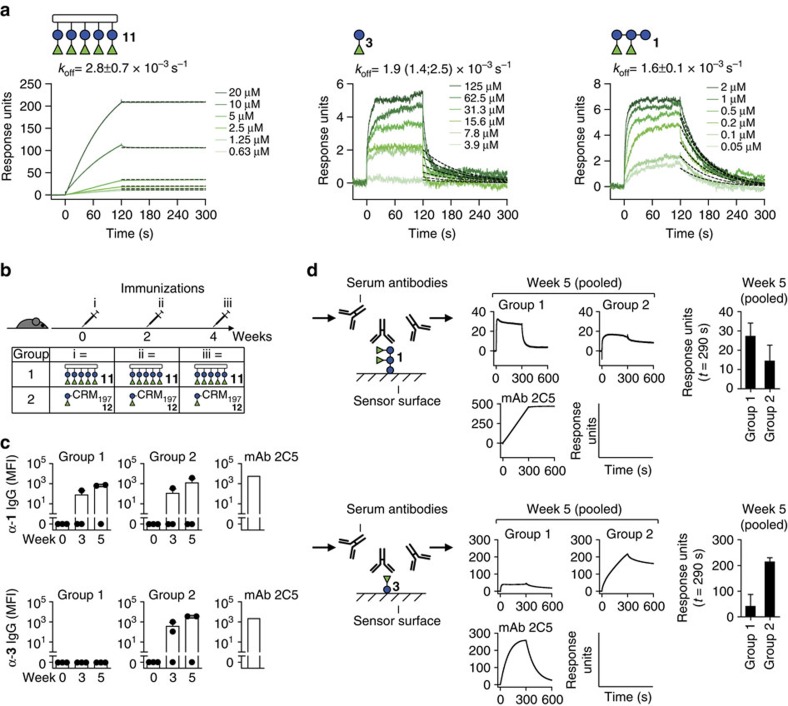
Antigenicity of multivalently displayed minimal glycan epitopes of PS-I. (**a**) SPR sensorgrams of mAb 2C5 binding to construct **11** that pentavalently presents the minimal disaccharide glycan epitope. For comparison, sensorgrams of the same antibody to disaccharide **3** and pentasaccharide **1** are shown. Avidities were estimated by fitting off-rate curves with a dissociation model (black dashed lines). The off-rate values *k*_off_ are mean±s.e.m. of *n*=3 or mean with minimum and maximum values in parentheses of *n*=2 independent measurements. (**b**) Mice were immunized with **11** (group 1) or glycoconjugate **12** (group 2) at the indicated time points. (**c**) Glycan microarray-inferred serum IgG levels to **1** (upper) and **3** (lower) of immunized mice expressed as mean fluorescence intensity (MFI) values. Bars represent mean+s.d. of three mice. Values of individual mice are shown as black dots. (**d**) SPR-inferred serum antibody responses to **1** (upper) and **3** (lower) in immunized mice. The experimental set-up is shown to the left. Average sensorgrams (two independent measurements) of pooled sera at week 5 post immunization subtracted by week 0 signals are shown in the centre. The bar graphs on the right show response unit signals at *t*=290 s (mean+s.e.m. of two independent measurements). See Supplementary Note 2 and Supplementary Figs 11–13 for details on the data presented in **b**–**d**.

First, we tested whether the thiol group influenced antibody binding using SPR. In line with the STD-NMR observations that the linker at the reducing end of **3** did not participate in antibody binding, **9** bound to mAb 2C5 with comparable kinetics and affinity (Supplementary Fig. 7b). The *K*_D_ values for **9** and **3** were highly similar (15.1 and 18 μM, respectively). Dose-dependent antibody binding to the monomeric construct **10** was also detected (Supplementary Fig. 7c). Owing to slow on-rates, perhaps resulting from steric constraints, binding curves did not reach equilibrium states, impeding *K*_D_ value calculation for **10**. However, the off-rates were similarly fast as observed for **3** and **9**. Slow on-rates were also seen for the pentavalent construct **11**–mAb interaction, but off-rates were considerably lower (Fig. 8a), indicating increased avidity to **11** compared with **10**.

To compare avidities, we used SPR to estimate the off-rates, which are the major determinants of antibody binding strength^13^,^32^,^33^,^34^,^35^,^36^,^37^. The off-rate of the **11**–mAb 2C5 interaction was 2.8 × 10^−8^ s^−1^ (Fig. 8a), whereas off-rates of **1**, **3** and **10** (Supplementary Fig. 7c) were about five orders of magnitude faster (1.9, 1.6 and 1.4 × 10^−3^ s^−1^, respectively). This demonstrated increased avidity to the pentameric compared with the monomeric construct. Tight antibody binding to OAA **11** demonstrated that antigenicity of larger PS-I structures could be mimicked by covalent linking of minimal glycan epitopes **3** on a synthetic scaffold.

### Compound 11 elicits antibodies to larger glycans

We next sought to investigate the ability of **11** to induce antibodies in mice. A group of mice was immunized with **11** three times in 2-week intervals (Fig. 8b). To be able to recruit T-cell helpers, **11** was equipped with a T-cell epitope encompassing amino acid residues 366–383 of the CRM_197_ protein^26^. Thereby, **11** represents a fully synthetic, multivalent vaccine candidate. For comparison, another group of mice was subjected to an identical immunization regime with glycoconjugate **12** composed of **3** and CRM_197_ (Supplementary Fig. 10a and Supplementary Note 2) that represents a semi-synthetic vaccine candidate. Serum IgG responses to the disaccharide **3** and pentasaccharide **1** were followed by glycan microarray (Fig. 8c) and SPR measurements (Fig. 8d). Most interestingly, **11** elicited IgG exclusively to **1**, whereas antibodies induced by **12** recognized both **3** and **1**. The latter observation confirmed previous results obtained with a comparable glycoconjugate of **3** and the CRM_197_ protein^7^. Both groups of mice also produced IgGs to mono-rhamnose **4**, as seen in our previous immunization studies with PS-I-CRM_197_ glycoconjugates^7^, and to CRM_197_, confirming the functionality of the synthetic CRM_197_ peptide in **11** (Supplementary Fig. 11). Although antibodies to CRM_197_ were detected in all mice after immunization, glycan-specific IgGs were only detected in one or two mice per group, indicating limited antigenicity of the glycan antigens (Fig. 8c). Next, we investigated whether the ability of **11** to elicit glycan-specific IgGs could be increased through its conjugation to CRM_197_, to furnish glycoconjugate **13** (Supplementary Fig. 10b and Supplementary Note 2). Although immunization of mice with **13** lead to glycan-specific IgGs to mono-rhamnose **4** only, an existing IgG response to **1** and **3** elicited by two immunizations of **12** could be boosted with **13**. Collectively, the immunization experiments showed that fully synthetic **11** was able to elicit IgGs to pentasaccharide **1** in mice at comparable levels to the semi-synthetic glycoconjugate **12**.

## Discussion

PS-I surface glycans are promising immunogens for vaccines against *C. difficile*^7^,^18^,^19^,^20^,^21^,^22^. We recently identified PS-I oligosaccharides that are recognized by antibodies of patients and therefore represent natural epitopes^7^. The disaccharide Rha-(1→3)-Glc **3** emerged as the smallest antigenic glycan and consequently provided a useful template for vaccines with low synthetic effort. However, its ability to raise antibodies in a conventional semi-synthetic glycoconjugate was limited^7^, calling for alternative means of presentation. Previous studies have shown that the multivalent display of small glycan antigens can yield highly immunogenic structures^38^. For instance, multivalently presented Tn monosaccharide antigens (α-*N*-acetylgalactosamine-serine) on dendrimeric^39^ or cyclic^40^ peptides were able to elicit high levels of anti-Tn antibodies in mice. This suggested that the multivalent display of PS-I disaccharides may lead to enhanced capability of inducing glycan-specific antibodies.

The feasibility of this approach required an understanding of how PS-I glycans interact with mammalian antibodies. Therefore, we studied glycan interactions with anti-PS-I mAbs using various biochemical and biophysical methods. Glycan microarray and SPR studies confirmed that disaccharide **3** is the minimal epitope of the PS-I glycan^7^. However, mAb binding to the more antigenic pentasaccharide **1** was stronger with nanomolar *K*_D_ values compared with **3** with micromolar *K*_D_ values. STD-NMR measurements revealed that mAb binding to PS-I glycans was primarily mediated by terminal rhamnoses and adjacent glucoses, but did not extend further into the antigens. The glycosidic bond that links two units of **3** in the pentasaccharide **1** does not directly participate in binding events. It may therefore be replaced by a linker to furnish structures that can mimic the immunologic properties of larger PS-I glycans. Molecular interaction studies indicated that stronger mAb binding to **1** was due to higher affinity, not avidity, despite the presence of two units of **3** in the pentasaccharide. ITC studies suggested that the entire pentasaccharide is accommodated by the binding pockets of the mAbs, leading to entropically favoured interactions. 2D NMR experiments showed that the pentasaccharide probably adopts a conformation with two disaccharide units in an angled position. Multivalently displayed disaccharides **3** might therefore lead to enhanced antibody binding through increased affinity (faster on-rates) when two adjacent disaccharides adopt a conformation similar to the pentasaccharide, through increased avidity (slower off-rates) by re-binding events, or a combination of both.

To investigate this, we synthesized OAAs multivalently presenting **3**. The OAA backbone has been shown to be non-toxic, non-immunogenic and suitable for the multivalent presentation of oligosaccharides with comparably low synthetic effort^23^,^24^,^25^. Straightforward solid-phase synthesis of OAAs furnished the PS-I glycan mimic **11** displaying five disaccharides **3**. This corresponds to 2.5 generic copies of the pentasaccharide, thereby enabling a combination of potential affinity- and avidity-enhancing effects. A comparably large construct was also chosen, as larger PS-I glycans tend to be more capable of eliciting antibodies than smaller ones^7^. Tight mAb binding to **11** was shown by SPR. Owing to slow on-rates that were outside of the measurable range, we were unable to detect whether affinity was increased. However, the off-rates were about five orders of magnitude slower than for monovalent disaccharides, indicating strong avidity effects. Thereby, we successfully created a PS-I glycan mimic showing high-avidity antibody binding. This is reminiscent of natural repetitive polysaccharides that enable strong antibody binding through avidity effects despite usually low affinities to individual epitopes^2^.

To investigate the ability of **11** to induce PS-I-specific antibodies, we performed immunization studies in mice. Compound **11** was equipped with a peptide epitope of CRM_197_ to recruit T-cell helpers, similar to previous immunization efforts with multivalent Tn antigens that included synthetic T-cell epitopes^39^,^40^. Antibody responses were compared with the semi-synthetic glycoconjugates **12** and **13** obtained by covalently linking **3** and **11**, respectively, to CRM_197_. The glycoconjugates were synthesized using a di-*p*-Nitrophenyl adipate ester spacer molecule^41^, to facilitate the challenging conjugation of **11** to CRM_197_, as this chemistry is more efficient than the previously^7^ used di-*N*-succinimidyl adipate ester.

Immunization of mice with fully synthetic OAA **11** induced IgGs to pentasaccharide **1** at low but comparable levels to semi-synthetic glycoconjugate **12**. Interestingly, in contrast to **12**, IgGs to disaccharide **3** were not detectable after immunization with **11**. Therefore, the IgG response to **11** was more specific to larger PS-I glycans, which is desirable for a vaccine to limit cross-reaction with structurally related glycans. This also indicated that two adjacent disaccharides in **11** may have adopted a conformation that resembles the pentasaccharide to some degree.

To investigate whether the ability of **11** to induce PS-I-specific antibodies could be enhanced, we conjugated the pentavalent construct to CRM_197_. The resulting glycoconjugate **13**, however, was unable to generate PS-I glycan-specific IgGs in mice, probably due to the low antigen loading of 1.3 mol of **11** per mole of CRM_197_. It has been noted previously that low antigen loading of glycoconjugates is associated with weaker antibody responses^42^. Glycoconjugate **13**, however, was able to boost existing IgG responses to PS-I glycans elicited by **12**, suggesting limited ability to raise antibodies that may be increased by higher antigen loading.

It has to be mentioned that the PS-I-specific IgG responses induced by **12**, displaying antigens at a high density (20 mol **3** per mole of CRM_197_) were relatively weak and only detectable at low serum dilutions (1:20) by glycan microarray. A comparable glycoconjugate with lower antigen density (10 mol **3** per mole of CRM_197_) investigated previously^7^ elicited higher levels of PS-I-specific IgGs in mice than **12** that were detectable at higher dilutions (1:100). It has been suggested previously that too high glycan loading of glycoconjugates may limit T-cell stimulation, resulting in weak antibody responses^42^. Higher antigen loading and therefore weaker IgG levels in the present study probably resulted from the employed conjugation chemistry. Still, glycoconjugate **12** was suitable to compare antigen recognition patterns to mice immunized with **11**, as **12** elicited IgGs cross-reacting to **1** and **3**, similar to our previous immunization studies^7^. Further studies are required to increase the ability of multivalently displayed disaccharides to elicit antibodies. As the linker itself is not involved in antibody binding, it may be replaced to alter the distance of disaccharide units, the biocompatibility and/or the flexibility of the construct. Comparative studies with constructs of different valencies may help to differentiate affinity from avidity effects. The antibody response to multivalent glycan mimics on immunization may also benefit from incorporation of immune stimulatory molecules such as glycosphingolipids, as we have shown recently^43^. Furthermore, the ability of the anti-PS-I antibodies to confer functional immunity requires investigation. Both active immunization and passive antibody transfer regimes are currently being tested for their ability to limit *C. difficile*-induced colitis in a murine challenge model. Chimerized and humanized versions of the mAbs will be designed that may be of potential therapeutic use.

In a broader sense, our findings provide insights into the nature of glycan–antibody interactions. Similar to lectins, antibody binding to glycan antigens is mainly an enthalpically driven process often of low millimolar to micromolar affinities^44^,^45^,^46^,^47^,^48^,^49^,^50^ and, in most cases, characterized by unfavourable entropic contributions^45^,^46^,^51^,^52^,^53^,^54^,^55^,^56^,^57^,^58^. Enthalpy–entropy compensation usually impedes high-affinity binding. Nanomolar affinities of antibodies against oligosaccharides are typically attributed to strong ionic interactions as in the case of a trisaccharide antigen of *Chlamydia*^59^,^60^ or to the conformational rigidity of larger polysaccharides that leads to favourable entropic contributions due to the lower entropic penalty during antibody binding^61^. In contrast, we here demonstrated antibodies binding with nanomolar affinity to a comparably small oligosaccharide antigen, pentasaccharide **1**, without charged residues. Details on these interactions were obtained by STD-NMR. This technique has been used to explain glycan selectivity of lectins at the molecular level^62^ and is also useful to decipher glycan–antibody interactions^8^. High-affinity binding to **1** could be due to antibody interactions with hydrophobic methyl groups, as shown by STD-NMR, which result in a favourable entropy by solvent displacement. Interestingly, although the number of mAbs in this study was limited, all three recognized the similar molecular epitope of PS-I mainly involving rhamnose. The notion that antibody affinity benefits from recognition of hydrophobic methyl groups in bacterial sugars is further supported by our recent observation that methyl groups of anthrose and rhamnose contribute significantly to nanomolar affinity antibody binding to a tetrasaccharide antigen of *Bacillus anthracis*^9^. The avidity-enhancing effect of presenting minimal glycan epitopes on a scaffold may also explain how repetitive bacterial polysaccharides can induce strongly binding antibodies, despite usually low affinities against single epitopes.

Overall, this study shows that the identification and multivalent presentation of minimal glycan epitopes can result in fully synthetic, highly antigenic glycan mimics that are able to elicit antibody responses specific for larger glycans. More generally, the findings advance our understanding of glycan–antibody interactions that are the basis for epitope-focused rational antigen design *en route* towards improved glycan-based vaccines against *C. difficile* and other pathogens expressing repetitive glycan antigens.

## Methods

### Preparation of glycan microarrays

Oligosaccharides bearing an amine-terminal linker, or proteins, were immobilized on *N*-hydroxyl succinimide ester-activated slides (CodeLink Activated Slides by SurModics, Inc., Eden Prairie, MN, USA) with a piezoelectric spotting device (S3; Scienion, Berlin, Germany) in such a way that 64 individual subarrays were contained each slide^27^. Microarray slides were incubated in a humid chamber for 24 h at room temperature (RT) to complete coupling reactions. Next, the remaining *N*-hydroxyl succinimide ester groups were quenched with 50 mM aminoethanol solution pH 9 for 1 h at 50 °C, washed three times with deionized water and stored desiccated until use, as described^27^.

### Generation of mAbs

mAbs to immunogen **1** were obtained by the hybridoma technique^27^ from mouse splenocytes immunized with a glycoconjugate composed of the CRM_197_ carrier protein (Pfénex, Inc., San Diego, CA, USA) and **1** synthesized with di-*N*-succinimidyl adipate as cross-linking reagent^7^. Six 6–8 weeks old female C57BL/6 mice (purchased from Charles River, Sulzfeld, Germany) were immunized with this glycoconjugate via the subcutaneous route in the presence of aluminium hydroxide adjuvant (Alum Alhydrogel, Brenntag) three times in 2-week intervals. Each immunization contained 3 μg of CRM_197_-bound immunogen **1**. The immune response was followed weekly by glycan microarray-assisted analysis of sera for IgG antibodies to **1**, CRM_197_ and the generic spacer moiety composed of aminopentyl and adipoyl moieties, and control oligosaccharides. For serum IgG analysis, printed and quenched microarray slides were blocked for 1 h with 1% (w/v) BSA in PBS, washed three times with PBS and dried by centrifugation (300 *g*, 5 min). Slides were then equipped with 64-well incubation chambers (FlexWell 64, Grace Bio-Labs, Bend, OR, USA) and incubated with mouse sera diluted 1:100 (v/v) in PBS for 1 h in a humid chamber. After washing three times with 0.1% Tween-20 in PBS (v/v) and drying by centrifugation, the microarray slides were incubated for 1 h with anti-mouse IgG Alexa Fluor 647 antibody (Life Technologies, catalogue number A-31574) diluted 1:400 in 1% BSA in PBS (w/v) in a humid chamber. After washing three times with 0.1% Tween-20 in PBS (v/v) and once with deionized water, microarray slides were dried by centrifugation and scanned with a GenePix 4300A microarray scanner (Molecular Devices, Sunnyvale, CA, USA). Of the six mice, the one with highest IgG response to **1** was selected for splenocyte isolation 1 week after the third immunization and splenocytes were fused with P3X63Ag8.653 myeloma cells (purchased from the American Type Culture Collection, Manassas, VA, USA), to obtain hybridomas. Hybridoma clones were selected by glycan microarray-assisted analysis^27^. After three subsequent subcloning steps, three hybridoma clones, 2C5, 10A1 and 10D6, producing IgGs exclusively to **1** were recovered.

### Purification and isotype analysis of mAbs

Hybridoma clones 2C5, 10A1 and 10D6 were expanded in serum-free medium as described^27^. Cell culture supernatants were concentrated tenfold using centrifugal filter devices with 50 kDa exclusion volume (Amicon Ultra-15 Ultracel, Millipore). Concentrated supernatants were subjected to IgG purification using the Proteus Protein G Antibody Purification Midi Kit (AbD Serotec) following the manufacturer's recommendations. Purified mAbs were stored in PBS with 0.02% (w/v) sodium azide at 4 °C. Protein concentrations were determined with the Pierce Micro BCA Protein Assay Kit (Thermo Scientific) and IgG isotypes were determined with the AbD Serotec Mouse Isotyping Kit MMT1 according to the manufacturer's recommendations.

### SDS-PAGE

Samples were dissolved in Lämmli buffer (0.125 M Tris, 20% (v/v) glycerol, 4% (w/v) SDS, 5% (v/v) β-mercaptoethanol and bromophenol blue pH 6.8) and boiled at 95 °C for 5 min. Samples were run in a 10% polyacrylamide gel and stained with 0.025% Coomassie Brilliant Blue R-250 in an aqueous solution containing 40% (v/v) methanol and 7% (v/v) acetic acid. Gel was destained with 50% (v/v) methanol and 10% (v/v) acetic acid in H_2_O. PageRuler Plus Prestained Protein Ladder (Thermo Scientific, catalogue number 26619) served as size marker.

### Glycan microarray binding assays

Microarray slides were blocked with 1% BSA in PBS (w/v) for 1 h at RT, washed three times with PBS and dried by centrifugation (300 *g*, 5 min). FlexWell 64 incubation chambers (Grace Bio-Labs) were applied to microarray slides. Slides were incubated with mouse sera diluted 1:100 with PBS unless mentioned otherwise, in a humid chamber for 1 h at RT, washed three times with 0.1% Tween-20 in PBS (v/v) and dried by centrifugation (300 *g*, 5 min). Slides were incubated with fluorescence-labelled secondary antibodies diluted 1:400 in 1% BSA in PBS (w/v) in a humid chamber for 1 h at RT, washed three times with 0.1% Tween-20 in PBS (v/v), rinsed once with deionized water and dried by centrifugation (300 *g*, 5 min) before scanning with a GenePix 4300A microarray scanner (Molecular Devices). Image analysis was carried out with the GenePix Pro 7 software (Molecular Devices). The photomultiplier tube voltage was adjusted such that scans were free of saturation signals. Background-subtracted mean fluorescence intensity values were exported to Microsoft Excel, for further analyses. Secondary antibodies used were as follows: Alexa Fluor 647 Goat Anti-Mouse IgG (H+L) (Life Technologies, catalogue number A-31574) and Alexa Fluor 594 Goat Anti-Mouse IgG1 (γ1) (Life Technologies, catalogue number A-21125).

### SPR and IM analysis

Binding analyses were carried out on a Biacore T100 instrument (GE Healthcare). CM5 sensor chips were functionalized with about 10,000 RUs of α-mouse IgG capture antibody, using the Mouse Antibody Capture Kit and the Amine Coupling Kit (GE Healthcare) according to the manufacturer's recommendations. A blank-immobilized flow cell was used as reference, to compensate for nonspecific binding of antibodies or oligosaccharides to the sensor chip surface. Kinetic measurements were performed with the Biacore T100 Control software using the ‘Kinetics' function. PBS was used as running buffer and all measurements were performed at 25 °C and a flow rate of 30 μl min^−1^. About 500 RUs of mAbs were captured at a concentration of 50 μg ml^−1^ diluted in PBS and oligosaccharides or OAAs (in PBS) at the indicated concentrations were passed through, using the standard parameters for association and dissociation times unless mentioned otherwise. Flow cells were regenerated with 10 mM glycine-HCl pH 1.7 for 30 s. Kinetic evaluation of binding responses was performed with the Biacore T100 Evaluation software, using reference-subtracted sensorgrams. Two different kinetic models, a 1:1 binding Langmuir model and a two-state reaction model, which assumes a conformational change in the antibody–analyte complex, were used to fit the data. The fitting quality was evaluated by investigating respective residual plots, *χ*^2^ and s.e. values for on- and off-rates. Both models yielded similar good fittings. We chose the 1:1 binding model over the two-state reaction model to calculate on- and off-rates, as well as *K*_D_ values, as this model makes fewer assumptions. When on-rates and/or off-rates were outside of the measurable ranges of the instrument, the steady-state affinity model was used instead to determine *K*_D_ values. The 1:1 binding model was generally preferred over the steady-state affinity model in cases where both could be applied. In these cases, the two models yielded roughly similar *K*_D_ values. Thermodynamic parameters were inferred by using the ‘Thermodynamics' function of the Biacore T100 Control software with the same experimental set-up described above. Temperatures from 13 to 37 °C were chosen. Values for Δ*G*, Δ*H* and Δ*S* were inferred by van't Hoff analysis. IM analysis was carried out by Ridgeview Diagnostics AB, Uppsala, Sweden.

### STD-NMR

Before the STD-NMR studies, proton resonances of oligosaccharides **1**–**3** were assigned using standard one-dimensional proton spectra, as well as 2D total correlation spectroscopy (2D-TOCSY), correlation spectroscopy, ^1^H–^13^C heteronuclear single quantum coherence and ^1^H–^13^C heteronuclear multiple bond correlation spectra at concentrations varying between 5 and 20 mM in D_2_O. All NMR studies were measured at 298 K on a Varian PremiumCOMPACT 600 MHz spectrometer equipped with a OneNMR probe. For STD-NMR, samples contained 200 μM ligand and 2 μM antibody in 40 mM phosphate buffer (pH 7.0, uncorrected). On-resonance irradiation was set to −0.5 p.p.m. and off-resonance irradiation was set to 80 p.p.m. A 35-ms T_1_ρ spin-lock filter and a W5 WATERGATE for solvent suppression were applied. The saturation pulse train consisted of a series of Gaussian shaped pulses of 50 ms duration and 1 ms interpulse delay with an irradiation power of 85 Hz. A total of 2,048 scans were recorded. STD-NMR spectra of carbohydrates in the absence of antibody and spectra of antibody only served as negative controls. Here, saturation and relaxation times were set to 2 and 6 s, respectively. STD build-up curves for mAb 10A1 were recorded at saturation transfer times of 0.5, 1, 2, 4 and 6 s, adjusting the prescan delay accordingly. Epitopes for mAbs 2C5 and 10D6 were recorded with a saturation transfer time of 2 s. NMR spectra were processed in MestReNova 9.1 (MestreLab) and data were analysed in OriginPro9 (OriginLab) to obtain the binding epitopes according to Mayer and James^28^. For the competition experiments, **1** was added to samples of **3** or **2** to a final concentration of 200 μM applying a saturation transfer time of 2 s.

### ITC

All measurements were performed in a MicroCal ITC200 system (GE Healthcare) at 25 °C. Oligosaccharides **1** and **3** (in PBS) at either 250 μM or 3 mM were titrated into the measurement cell containing 7 μM of mAb 2C5 or 10A1 in PBS (injection volume 2 μl). Data analysis was performed with the OriginPro 8.6G software (MicroCal) provided with the instrument, using the one set of site model to fit the data points to infer thermodynamic parameters and stoichiometry values. For the low *c*-value measurements for **3**, the stoichiometry was set constant to 2.

### Conformation of **1** as determined by NMR and modelling

Solution conformation of **1** was investigated by 2D-TOCSY and NOE spectroscopy NMR experiments. Compound **1** was dissolved in D_2_O at 5.7 mM and spectra were obtained at 298 K. The NOE spectroscopy spectrum was acquired at 500 ms mixing time, 512 increments in f1 at 16 scans per increment with an acquisition time of 0.57 s, an inter-scan delay of 2 s and a zero-quantum filter. The corresponding zTOCSY was acquired with the same settings as above using four scans and a DIPSI2 spinlock with a mixing time of 120 ms. Data were processed in MestreNova 9.0 (MestreLab). Solution structure of **1** was calculated using GLYCAM06 force field^31^ and analysed with UCSF Chimera package^63^.

### Synthesis of oligosaccharide-functionalized OAAs

OAA building block synthesis and solid phase assembly of the oligomeric backbone was performed following previously established protocols^25^. Conjugation of **9** was performed in batch starting from a solution of acetonitrile, water, acetic acid, radical initiator and tris(2-carboxyethyl)phosphine, which was carefully degassed and added to lyophilized oligomer precursors and **9**. Conjugation reactions were performed in a standard transparent HPLC vial (1.5 ml) and were irradiated for 18 h using an ExoTerra ReptiGlo 5.0 UVB 26 W terrarium lamp (compound **10**, radical initiator: benzophenone) or a Heraeus TQ 150, 150 W medium-pressure mercury lamp (compound **11**, radical initiator: 4,4-Azobis(4-cyanovaleric acid))^64^. After ultraviolet irradiation, the reaction mixture was purified by reversed-phase HPLC (compound **10**) or size-exclusion chromatography using Sephadex G-25 (compound **11**). The resulting conjugates were obtained as partially or fully oxidized sulfoxide-linked products (Supplementary Fig. 9). Reversed-phase HPLC chromatograms of **10**, **11** and their precursors are shown in Supplementary Fig. 14. Matrix-assisted laser desorption/ionization–time of flight mass spectra of both precursors are shown in Supplementary Fig. 15. A short peptide sequence comprising amino acids 366–383 of the CRM_197_ protein^26^ was introduced to recruit T-cell helpers for immunization experiments.

### Preparation of glycoconjugates **12** and **13**

Conjugation reactions followed a protocol modified from Wu *et al*.^41^ In brief, to obtain **12**, 2.2 mg of disaccharide **3** (5.3 μmol) were reacted with sixfold molar excess of homobifunctional adipic acid *p*-nitrophenyl ester (see Supplementary Fig. 10a) in dimethyl sulfoxide/pyridine (2:1) in the presence of Et_3_N for 2 h at RT. Excess linker was removed by washing with dichloromethane/diethylether (1:1). This yielded 2.6 mg the half ester shown in Supplementary Fig. 10a (74% yield). A quantity of 1.4 mg (2.1 μmol) of half ester were reacted with 2 mg (34.2 nmol) of CRM_197_ (Pfénex) in 100 mM sodium phosphate pH 8 for 24 h at RT. To obtain **13**, 380 μg of construct **11** (71.3 nmol) were reacted with sixfold molar excess of spacer in dimethyl sulfoxide/pyridine (2:1) in the presence of Et_3_N for 2 h at RT. Excess linker was removed by washing with dichloromethane/diethylether (1:1). This yielded 230 μg of the half ester shown in Supplementary Fig. 10b (58% yield). Two hundred and thirty micrograms of this half ester were reacted with 0.5 mg (8.6 nmol) of CRM_197_ in 100 mM sodium phosphate pH 8 for 24 h at RT. After the reactions, both glycoconjugates were desalted and concentrated with ddH_2_O, using centrifugal filter devices with an exclusion volume of 10,000 Da (Amicon Ultracel, Millipore). Protein concentrations were determined by measuring the absorbance at a wavelength of 280 nm in a Nanodrop ND-1000 spectrophotometer (Thermo Fisher Scientific), employing an extinction coefficient of 54,320 M^−1^ cm^−1^.

### Western blottings

Proteins were separated by SDS–PAGE as described above and electroblotted onto polyvinylidene difluoride membranes. Ponceau S staining was performed to confirm successful transfer. After washing to remove the Ponceau stain, polyvinylidene difluoride membranes were blocked with TBS-T (Tris-buffered saline with 0.05% (v/v) Tween-20) supplemented with 5% (w/v) skimmed milk powder. The CRM_197_ protein was immunolabelled with goat anti-diphtheria toxin antibody (Abcam, catalogue number ab19950) diluted 1:2,500 in TBS-T with 1% (w/v) BSA and detected with anti-goat IgG horseradish peroxidase conjugate antibody (Sigma-Aldrich, catalogue number A4174) diluted 1:5,000 in TBS-T with 1% (w/v) BSA after three washing steps with TBS-T. PS-I glycans were immunolabelled with a mixture of mAbs 2C5, 10A1 and 10D6, each at 2.5 μg ml^−1^ in TBS-T with 1% (w/v) BSA and detected with anti-mouse IgG horseradish peroxidase conjugate antibody (Dianova, catalogue number 115-035-062) diluted 1:10,000 in TBS-T with 1% (w/v) BSA after three washing steps with TBS-T. Chemoluminescence was detected using the Amersham ECL Western Blotting Detection Reagent (GE Healthcare) according to the manufacturer's recommendations, in a LAS-4000 imager (Fujifilm).

### Carbohydrate concentration determination by anthrone assay

Anthrone reactions followed a modified protocol by Turula *et al*.^65^ To 35 μl of a 0.1% (w/v) solution of anthrone reagent (Sigma) in concentrated (98%) sulfuric acid, 5 μl of desalted solutions of **12**, **13** or CRM_197_ were added in 96-well round-bottom microtitre plates. Absorbance at 579 nm was determined in a spectrophotometric plate reader and compared with standard curves using equimolar amounts of D-glucose and L-rhamnose. Such, average antigen-to-CRM_197_ molar ratios were calculated. For **12** and **13**, this yielded 20 mol of **3** and 1.3 mol of **11** (equal to 6.5 mol of **9**) per mole of CRM_197_, respectively.

### Immunizations

Six- to eight-week-old female C57BL/6 mice were purchased from Charles River. Mice were immunized subcutaneously with different immunization regimes three times in 2-week intervals with an amount of construct **11** corresponding to 5 μg glycan antigen or glycoconjugates **12** and **13** (1 μg glycan antigen). Three mice per group were chosen, as this study aimed to qualitatively assess whether compounds **11**–**13** were capable of eliciting antibodies, without intention of quantitatively comparing antibody levels. Therefore, no randomization or blinding was required. Freund's Adjuvant (Sigma) was used for all immunizations according to the manufacturer's recommendations. Complete Freund's Adjuvant was used for initial immunizations at week 0 and Incomplete Freund's Adjuvant was used for subsequent immunizations at weeks 2 and 4. Animal experiments were approved by the Landesamt für Gesundheit und Soziales, Berlin, and performed in strict accordance with the German regulations of the Society for Laboratory Animal Science and the European Health Law of the Federation of Laboratory Animal Science Associations. All efforts were made to minimize suffering.

### Determination of serum antibody responses by SPR

Antibody binding analyses were carried out on a Biacore T100 instrument (GE Healthcare). Flow cells of a CM5 sensor chip were immobilized with 1 mM solutions of pentasaccharide **1** (final response 390.5 RU) or disaccharide **3** (247.5 RU) in 100 mM sodium phosphate buffer pH 8.5, using the Amine Coupling Kit (GE Healthcare) according to the manufacturer's recommendations. Flow cells immobilized with about 10,000 RU of BSA served as reference. Kinetic measurements with mouse sera diluted 1:100 in PBS were performed with the Biacore T100 Control software, using the standard parameters of the ‘Kinetics' function but with extended contact and dissociation times of 300 s. PBS was used as running buffer and all measurements were performed at 25 °C and a flow rate of 30 μl min^−1^. Binding signals of pooled (*n*=3 mice) post-immunization (week 5) sera were subtracted by pre-immunization (week 0) sera signals. Binding curves were extracted for further analysis in Microsoft Excel. The binding signals at *t*=290 s contact time served as antibody binding signal.

## Additional information

**How to cite this article:** Broecker, F. *et al*. Multivalent display of minimal *Clostridium difficile* glycan epitopes mimics antigenic properties of larger glycans. *Nat. Commun.* 7:11224 doi: 10.1038/ncomms11224 (2016).

## Supplementary Material

Supplementary Information Supplementary Figures 1-15, Supplementary Table 1, Supplementary Notes 1-2 and Supplementary References

## Figures and Tables

**Figure 1:**
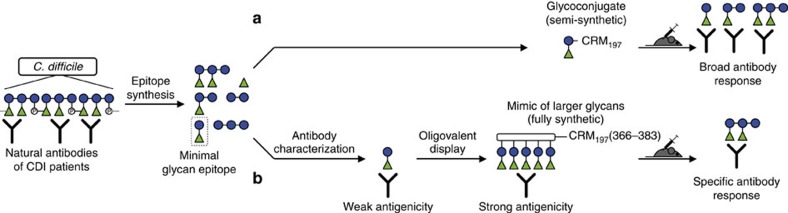
Identifying and connecting minimal glycan epitopes to mimic larger glycans. During *C. difficile* infections (CDIs), patients mount antibodies to the PS-I polysaccharide. In efforts towards rationally designed vaccines, various PS-I glycan epitopes were synthesized^7^. A disaccharide minimal epitope (dashed lines) was identified from recognition patterns of human and mouse anti-PS-I antibodies^7^. (**a**) Mice immunized with a semi-synthetic glycoconjugate vaccine candidate of CRM_197_ and the disaccharide produce antibodies to the pentasaccharide repeating unit and smaller substructures. (**b**) A fully synthetic pentavalent glycan mimic with increased antigenicity compared with monovalent disaccharides elicits antibodies to the pentasaccharide only. It comprises an OAA backbone^23^,^24^,^25^ and a T-cell epitope, amino acids 366–383 of the CRM_197_ protein^26^.

**Table 1:** SPR-inferred *K*_D_ values of anti-PS-I mAbs to PS-I oligosaccharide antigens.

	*K*_D_ values to oligosaccharide antigens				
mAb	1	2	3	4	6
2C5	163±23 nM	14.1 (10.3;17.8) μM	18.0 (16.3;19.6) μM	>>160 μM	>>160 μM
10A1	245 (211;280) nM	13.3 (13.0;13.6) μM	5.9 (5.3;6.4) μM	>>160 μM	>>160 μM
10D6	169 (128;209) nM	18.8 (17.2;20.3) μM	37.5±0.4 μM	>>160 μM	>>160 μM

mAb, monoclonal antibody; PS-I, polysaccharide-I; SPR, surface plasmon resonance.

See Supplementary Figs 3–5 for details. All values are mean±s.e.m. of *n*=3 or mean with minimum and maximum values in parentheses of *n*=2 independent experiments.
