# Supplementary material for: Multivalent display of minimal Clostridium difficile glycan epitopes mimics antigenic properties of larger glycans
Source: Nat Commun. 2016 Apr 19;7:11224. doi: 10.1038/ncomms11224 (PMC4838876; doi:10.1038/ncomms11224)
Supplement: Supplementary Information — Supplementary Figures 1-15, Supplementary Table 1, Supplementary Notes 1-2 and Supplementary References [file ncomms11224-s1.pdf]

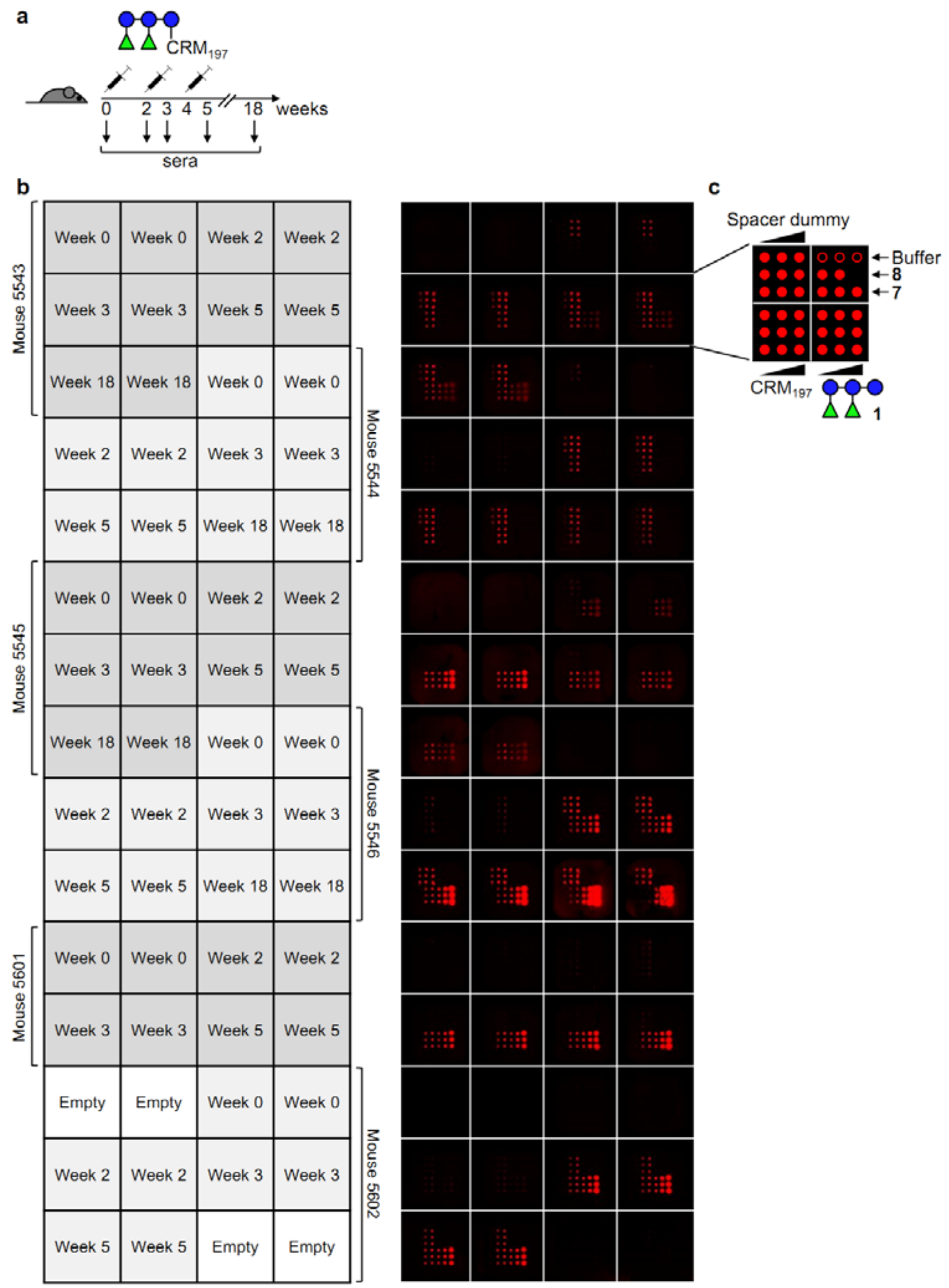

Supplementary Figure 1 | Microarray analysis of serum IgG antibodies of mice immunized with pentasaccharide 1-CRM$_{197}$ glycoconjugate. (**a**) Immunization regime. (**b**) Microarray scan. Sample layout is shown to the left. Mouse numbers 5543 through 5602 are indicated. Fluorescent spots excited with a 635 nm laser of the microarray slide incubated with mouse sera (1:100 dilution in PBS) and subsequently with Alexa Fluor 647 Goat Anti-Mouse IgG (H+L) antibody (Life Technologies; 1:400 dilution in PBS with 1 % (w/v) BSA) are shown. (**c**) Printing pattern of the microarray slide (see Fig. 3b in the main manuscript for details). BSA-Spacer-GlcNAc is a dummy conjugate prepared with the same chemistry as the pentasaccharide **1**-CRM$_{197}$ glycoconjugate. Buffer is 50 mM sodium phosphate, pH 8.5. Mouse 5601 was selected for splenectomy and hybridoma cell fusion.

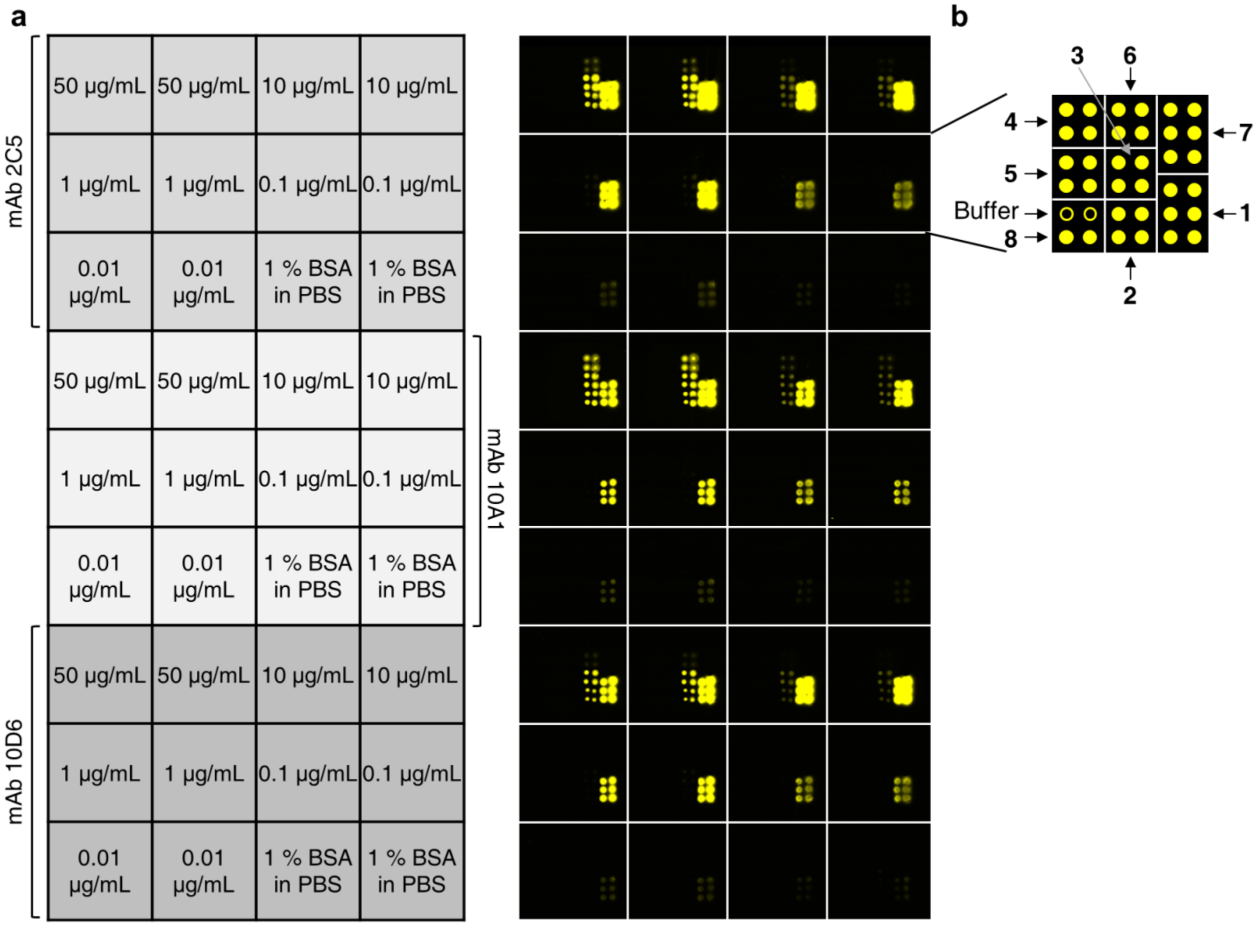

Supplementary Figure 2 | Microarray analysis of three purified mAbs against *C. difficile* PS-I. (**a**) Microarray scan. Sample information is shown to the left. Three purified mAbs, 2C5, 10A1 and 10D6, were incubated on the microarray slide at different concentrations in PBS with 1 % (w/v) BSA. Fluorescent spots excited with a 594 nm laser of the microarray slide incubated with mAbs and subsequently with Alexa Fluor 594 Goat Anti-Mouse IgG1 (γ1) antibody (Life Technologies; 1:400 dilution in PBS with 1% (w/v) BSA) are shown. (**b**) Printing pattern of the microarray slide (see Fig. 4c in the main manuscript for details). Buffer is 50 mM sodium phosphate, pH 8.5.

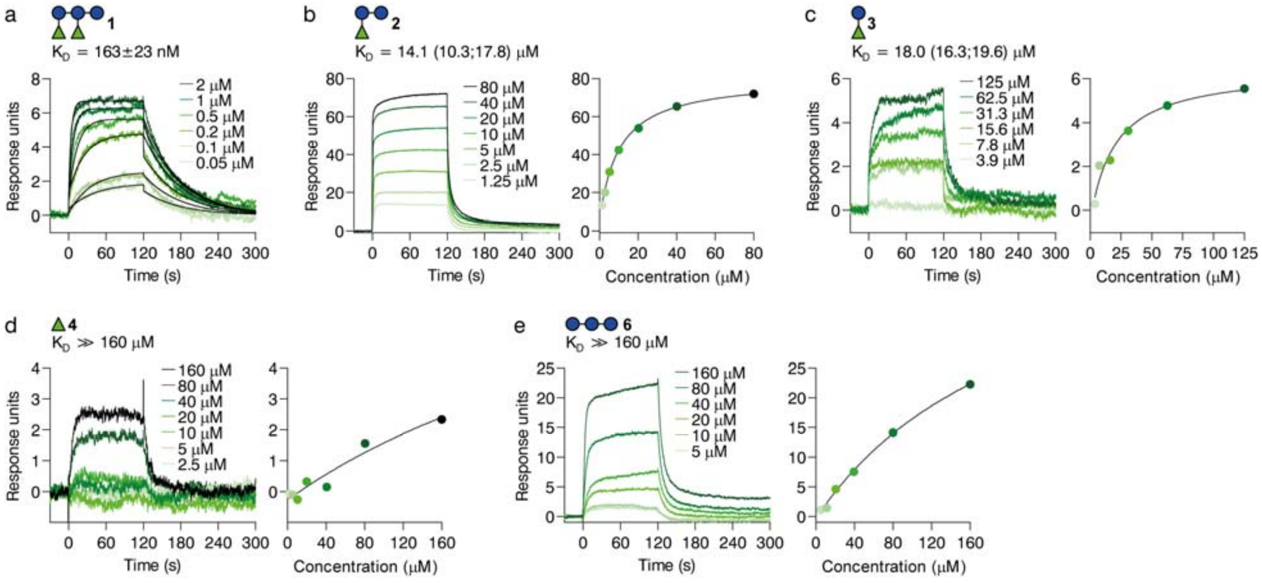

Supplementary Figure 3 | K_D values of mAb 2C5 to PS-I oligosaccharide antigens inferred by SPR. The sensor surface of a CM5 surface plasmon resonance chip was immobilized with about 10,000 response units of anti-mouse IgG capture antibody (Mouse Antibody Capture Kit, GE Healthcare). mAbs were captured and glycan antigens were passed through the sensor chip to monitor changes in the response unit signals (see legend of Fig. 5a in the main manuscript). Representative sensorgrams showing reference-subtracted binding signals to the PS-I pentasaccharide **1** (**a**), trisaccharide **2** (**b**), disaccharide **3** (**c**), mono-rhamnose **4** (**d**) and triglucose **6** (**e**) are shown. For **1**, the K_D value was inferred with a 1:1 Langmuir binding model (black overlaid curves). For **2** and **3**, K_D values were inferred with a steady-state affinity model. For **4** and **6**, saturation was not achieved in the tested concentration range, indicating K_D values were above 160 μM. All sensorgrams were recorded on a Biacore T100 instrument (GE Healthcare) using standard parameters of the "Kinetics" function. The indicated K_D values are mean ± s.e.m. of n=3 or mean with minimum and maximum values in parentheses of n=2 independent measurements.

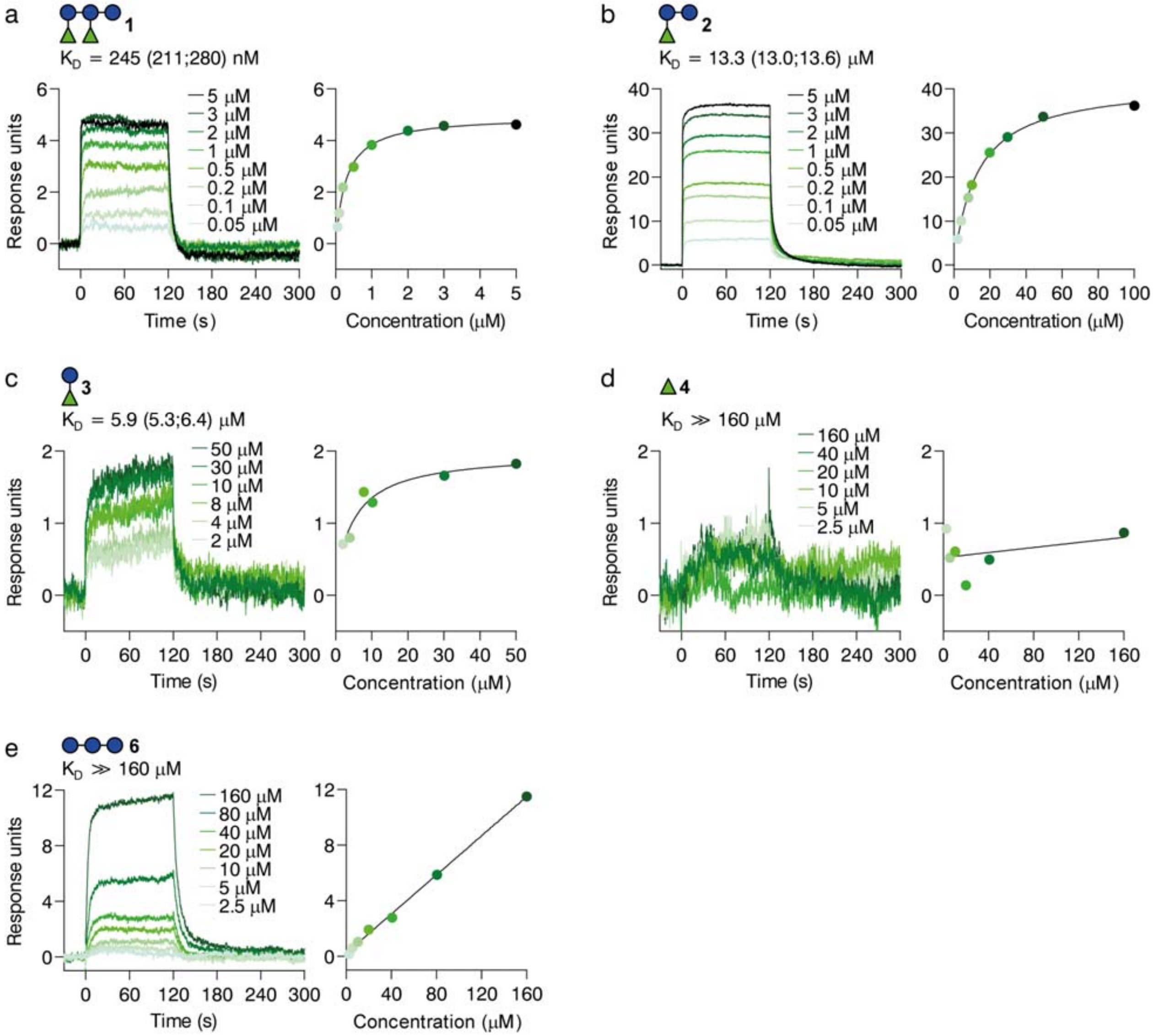

Supplementary Figure 4 | K_D values of mAb 10A1 to PS-I oligosaccharide antigens inferred by SPR. The sensor surface of a CM5 surface plasmon resonance chip was immobilized with about 10,000 response units of anti-mouse IgG capture antibody (Mouse Antibody Capture Kit, GE Healthcare). mAbs were captured and glycan antigens were passed through the sensor chip to monitor changes in the response unit signals (see legend of Fig. 5a in the main manuscript). Representative sensorgrams showing reference-subtracted binding signals to the PS-I pentasaccharide **1** (**a**), trisaccharide **2** (**b**), disaccharide **3** (**c**), mono-rhamnosyl **4** (**d**) and triglucosyl **6** (**e**) are shown. For **1-3**, the K_D values were inferred with a steady-state affinity model. For **4** and **6**, saturation was not achieved in the tested concentration range, indicating K_D values were above 160 μM. All sensorgrams were recorded on a Biacore T100 instrument (GE Healthcare) using standard parameters of the "Kinetics" function. The indicated K_D values are mean with minimum and maximum values in parentheses of n=2 independent measurements.

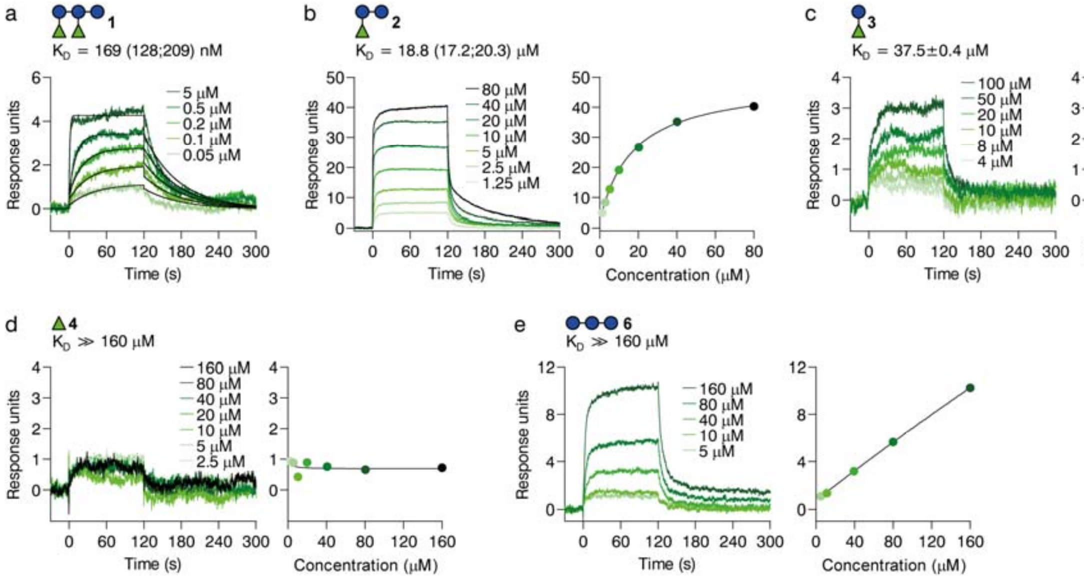

Supplementary Figure 5 | K_D values of mAb 10D6 to PS-I oligosaccharide antigens inferred by SPR. The sensor surface of a CM5 surface plasmon resonance chip was immobilized with about 10,000 response units of anti-mouse IgG capture antibody (Mouse Antibody Capture Kit, GE Healthcare). mAbs were captured and glycan antigens were passed through the sensor chip to monitor changes in the response unit signals (see legend of Fig. 5a in the main manuscript). Representative sensorgrams showing reference-subtracted binding signals to the PS-I pentasaccharide **1** (**a**), trisaccharide **2** (**b**), disaccharide **3** (**c**), mono-rhamnosyl **4** (**d**) and triglucosyl **6** (**e**) are shown. For **1**, the K_D value was inferred with a 1:1 Langmuir binding model (black overlaid curves). For **2** and **3**, K_D values were inferred with a steady-state affinity model. For **4** and **6**, saturation was not achieved in the tested concentration range, indicating K_D values were above 160 μM. All sensorgrams were recorded on a Biacore T100 instrument (GE Healthcare) using standard parameters of the "Kinetics" function. The indicated K_D values are mean ± s.e.m. of n=3 or mean with minimum and maximum values in parentheses of n=2 independent measurements.

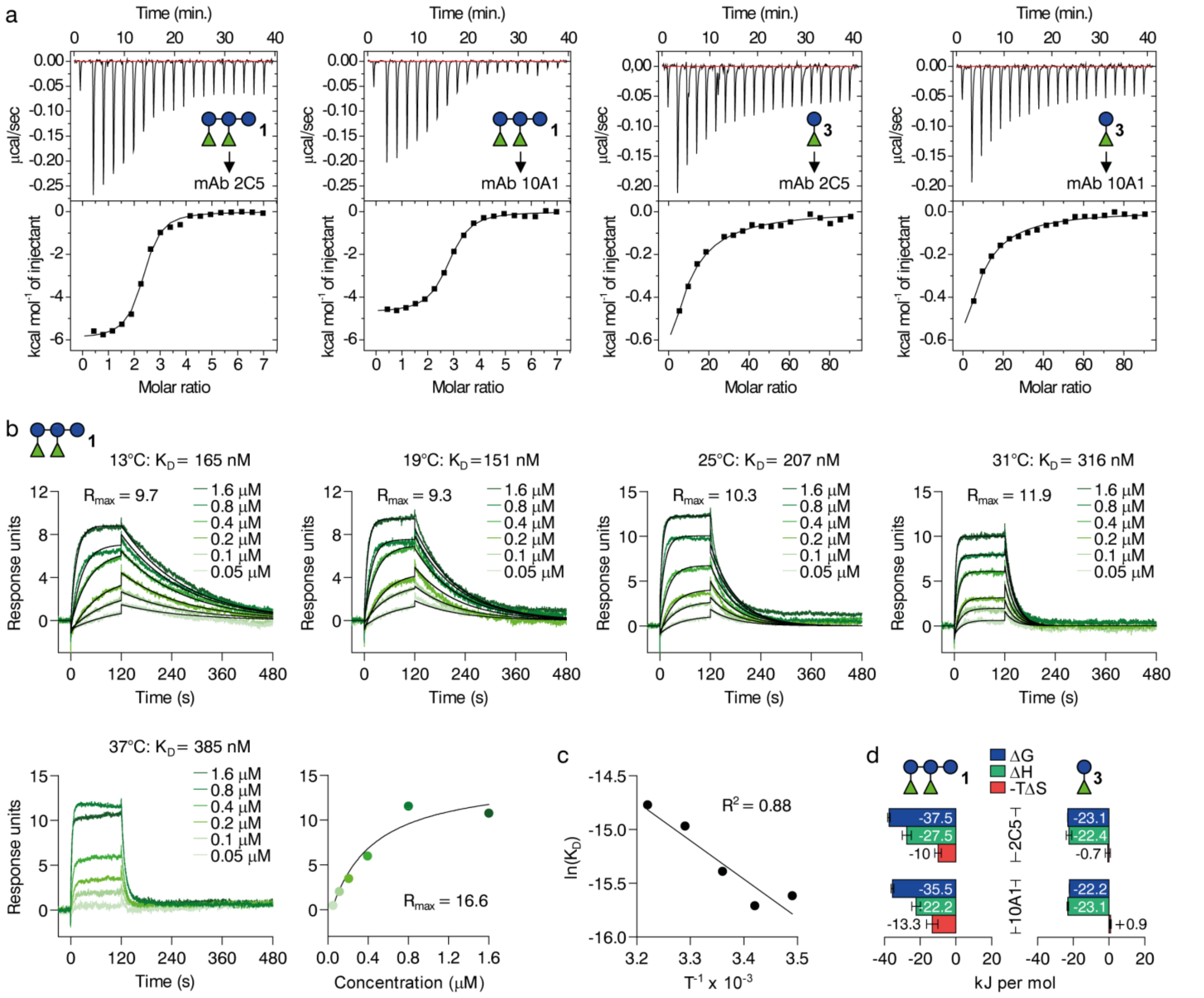

Supplementary Figure 6 | Thermodynamic parameters of PS-I-antibody interactions. (**a**) Thermograms recorded on a MicroCal iTC200 system (GE Healthcare) using 7 µM of mAb 2C5 or 10A1 and 0.25 mM of pentasaccharide **1** or 3 mM of disaccharide **3** as injectant at 25°C. Buffer was PBS and injection volume was 2 µL. Thermodynamic parameters were inferred by non-linear least square fits of the data points. For the interactions with disaccharide **3**, binding stoichiometries were set constant to 2. (**b**) Sensorgrams representing SPR measurements of mAb 2C5 interacting with pentasaccharide **1** at different temperatures. Except for the measurement at 37°C, K_D values were inferred by fitting of the data with a 1:1 Langmuir binding model (black overlaid curves). The binding curves of the measurement at 37°C could not be fitted with this model, and therefore, this K_D value was inferred by a steady-state affinity model instead. (**c**) Van-'t-Hoff plot of the data presented in **b**. From this plot, the following thermodynamic parameters were calculated: ΔH = -30 kJ per mol, -TΔS = -8.1 kJ per mol, ΔG = -38.1 kJ per mol. (**d**) Summary of thermodynamic parameters for both antigens interacting with mAbs 2C5 and 10A1. Bars represent mean ± s.e.m. of two independent ITC measurements.

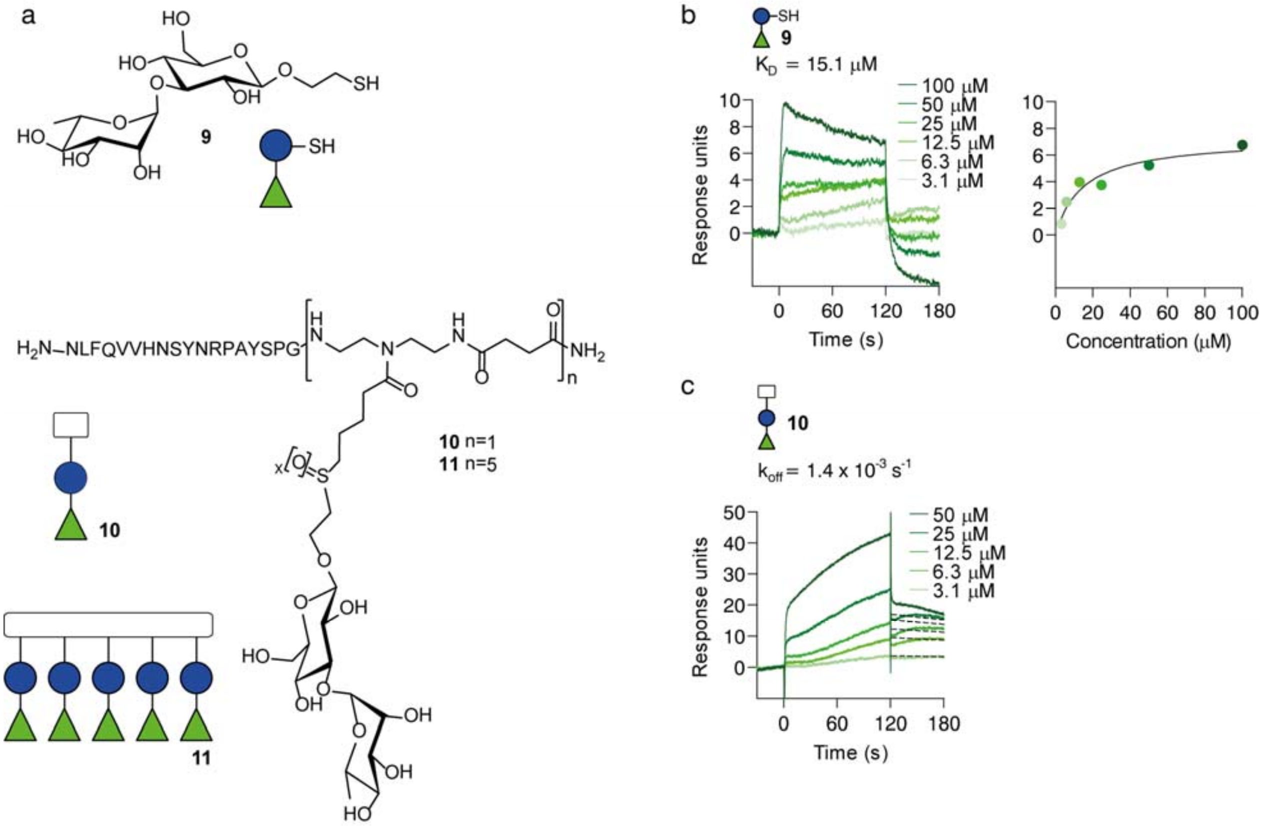

Supplementary Figure 7 | Structures of compounds 9-11 and affinities of mAb 2C5 binding to 9 and 10 inferred by SPR. (**a**) Chemical structures of compounds **9-11**. (**b**) and (**c**) Sensorgrams of mAb 2C5 binding to **9** (**b**) or to **10** (**c**). The sensor surface of a CM5 surface plasmon resonance chip was immobilized with about 10,000 response units of anti-mouse IgG capture antibody (Mouse Antibody Capture Kit, GE Healthcare). mAb 2C5 was captured and **9** or **10** was passed through the sensor chip to monitor changes in the response unit signals (see legend of Fig. 5a in the main manuscript). Affinity of **10** was estimated by fitting the off-rate curves with a dissociation model (overlaid black dashed lines). The synthesis of **9** is described in Supplementary Fig. 8. The synthesis of **10** and **11** is described in Supplementary Fig. 9.

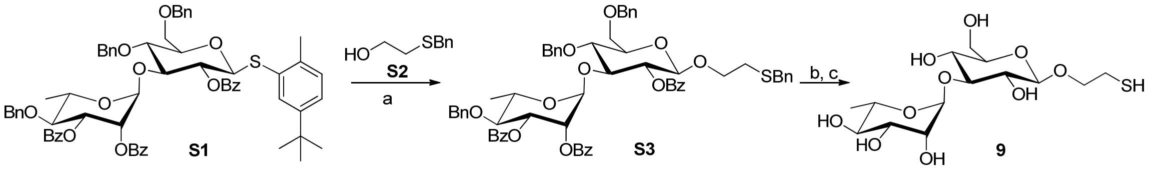

Supplementary Figure 8 | Synthesis of disaccharide 9. Reagents and conditions: a) DMTST, TTBPy, 3Å MS, DCM, 0°C to rt, 65%; b) NaOMe, MeOH; c) NH_3(liquid), THF, *t*-BuOH, Na, -78°C 81% over two steps.

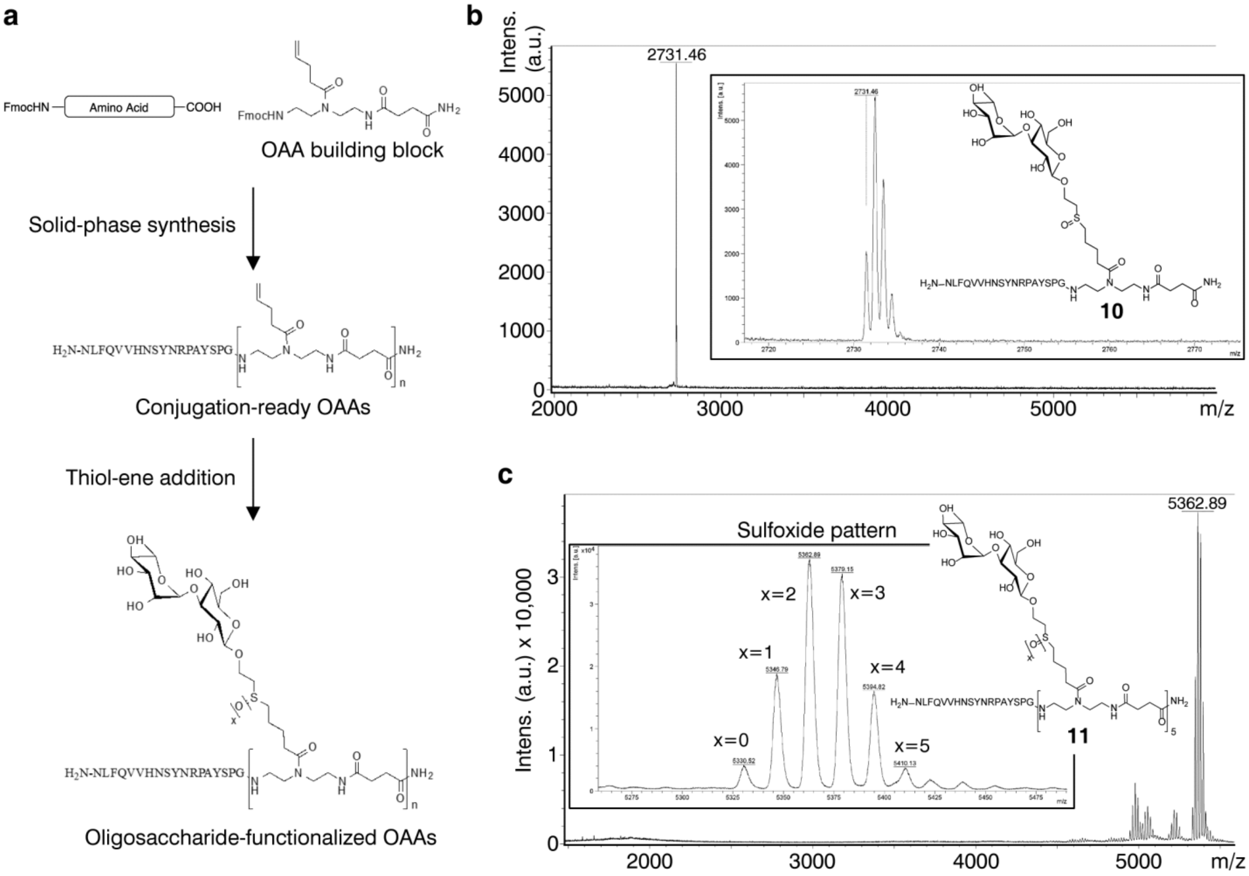

Supplementary Figure 9 | Synthesis scheme and MALDI analysis of 10 and 11. (**a**) Synthetic strategy towards oligosaccharide-functionalized oligo(amidoamines) (OAAs) **10** and **11**: Fmoc solid phase peptide and OAA synthesis were combined to obtain conjugation-ready, double-bond presenting OAAs, which were then further functionalized with disaccharide **9** using orthogonal thiol-ene addition; (**b**) MALDI-TOF-MS spectra of studied OAA conjugate **10**: *m/z* calcd $[M+H]^+$, 2731.39 (monoisotopic); found 2731.46; (**c**) MALDI-TOF-MS spectra of studied OAA conjugate **11**: *m/z* (sulfoxide pattern x = 0-5) calcd for x = 0 $[M+H]^+$, 5331.95 (average); found 5330.52; calcd for x = 1 $[M+H]^+$, 5347.95 (average); found 5346.79; calcd for x = 2 $[M+H]^+$, 5363.95 (average); found 5362.89; calcd for x = 3 $[M+H]^+$, 5379.95 (average); found 5379.15; calcd for x = 4 $[M+H]^+$, 5395.95 (average); found 5394.82; calcd for x = 5 $[M+H]^+$, 5411.99 (average); found 5410.13.

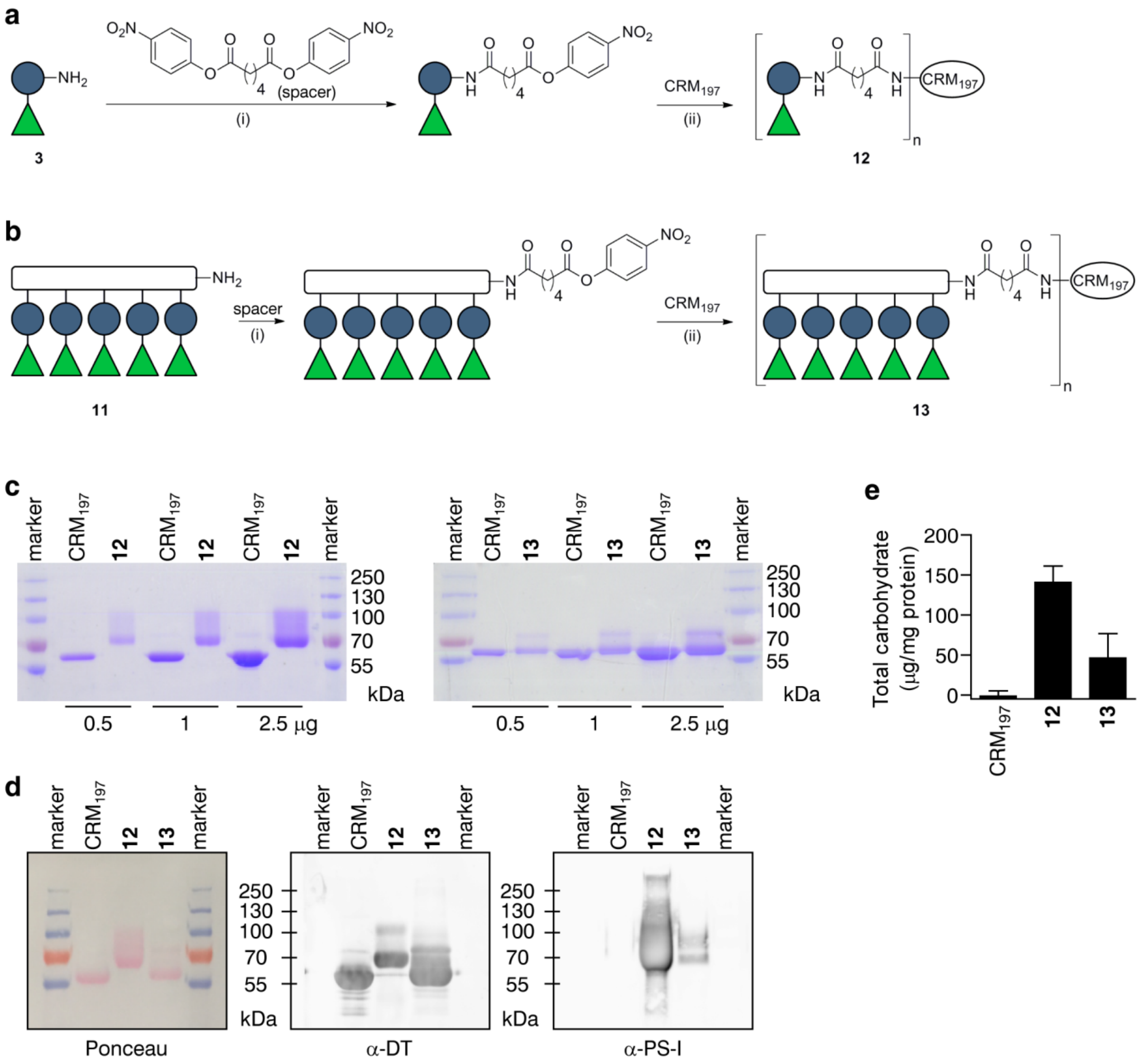

Supplementary Figure 10 | Synthesis and characterization of glycoconjugates 12 and 13. (**a**,**b**) Reaction schemes to obtain glycoconjugates **12** (**a**) and **13** (**b**). Reagents and conditions: (i) DMSO/pyridine, Et_3N, 2 h, room temperature; (ii) 100 mM sodium phosphate, pH 8, 24 h, room temperature. The reaction conditions were modified from Wu *et al.*[1]. (**c**) Coomassie-stained SDS-PAGE gels of **12** (left) and **13** (right). Non-conjugated carrier protein CRM_{197} was loaded for comparison. Marker band sizes in kDa are indicated. (**d**) Western Blots of glycoconjugates **12** and **13** and CRM_{197}. 2.5 µg protein was loaded in each lane. Marker band sizes in kDa are indicated. α-DT, anti-diphtheria toxin antibody; α-PS-I, anti-PS-I antibodies. (**e**) Quantification of total carbohydrate contents of glycoconjugates and CRM_{197} inferred by anthrone assays. Bars show mean + s.e.m. of two independent measurements.

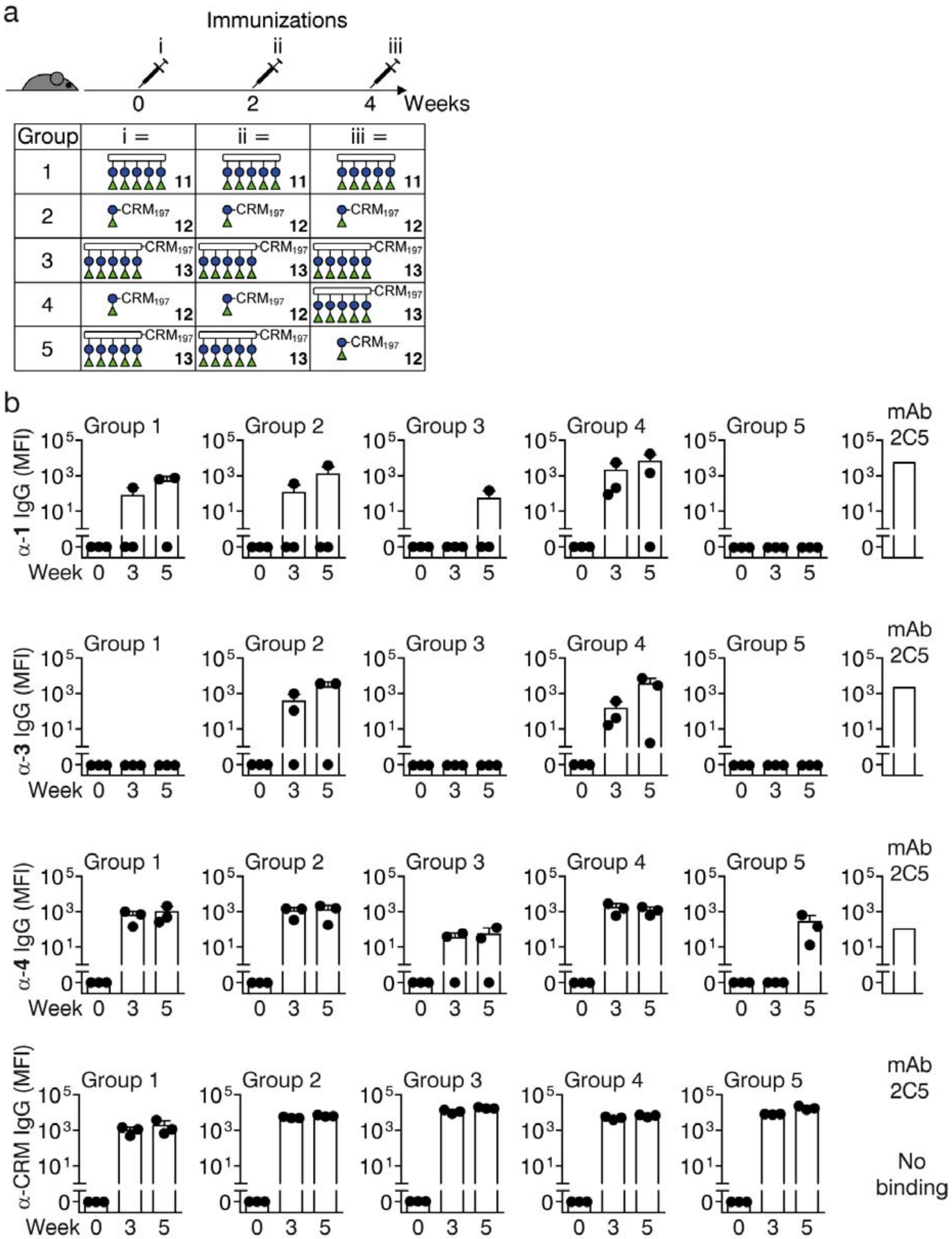

Supplementary Figure 11 | Glycan microarray-based detection of IgG responses in mice immunized with 11, 12, and/or 13. (**a**) Mice were immunized with the indicated regimes in two-week intervals. (**b**) Glycan array-inferred mean fluorescence intensity (MFI) values representing serum IgG levels to the indicated antigens. Black dots represent individual mice and bars represent mean + SD of three mice. Binding signals of mAb 2C5 at 10 µg mL^{-1} are shown as controls. See Supplementary Fig. 13 for representative microarray scans.

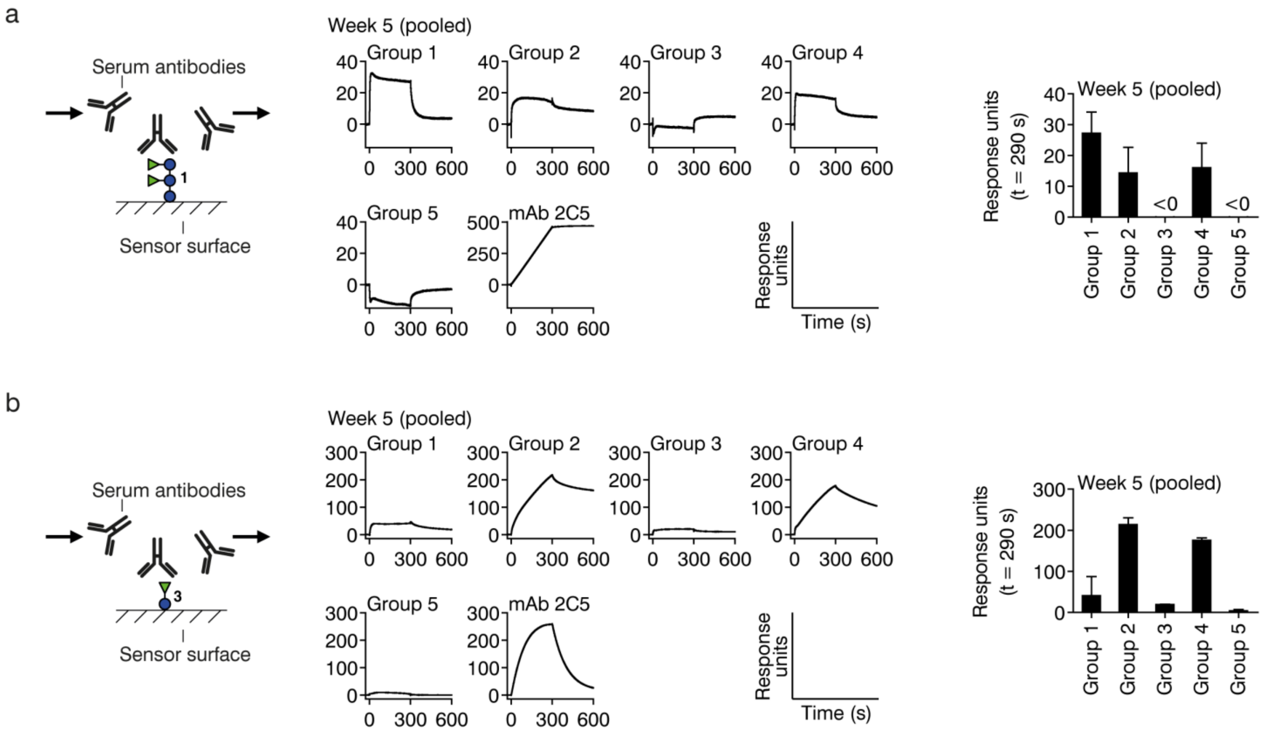

Supplementary Figure 12 | SPR-based detection of antibody responses in mice immunized with 11, 12 and/or 13. SPR-inferred serum antibody responses to **1** (**a**) and **3** (**b**) in immunized mice (see Supplementary Fig. 11a for a description of the mouse groups). The experimental set-up is shown to the left. Average sensorgrams (two independent measurements) of pooled sera at week 5 post-immunization subtracted by week 0 signals are shown in the center, with mAb 2C5 at 10 μg mL^{-1} as control. The bar graphs on the right show response unit signals at t = 290 s (mean + s.e.m. of two independent measurements).

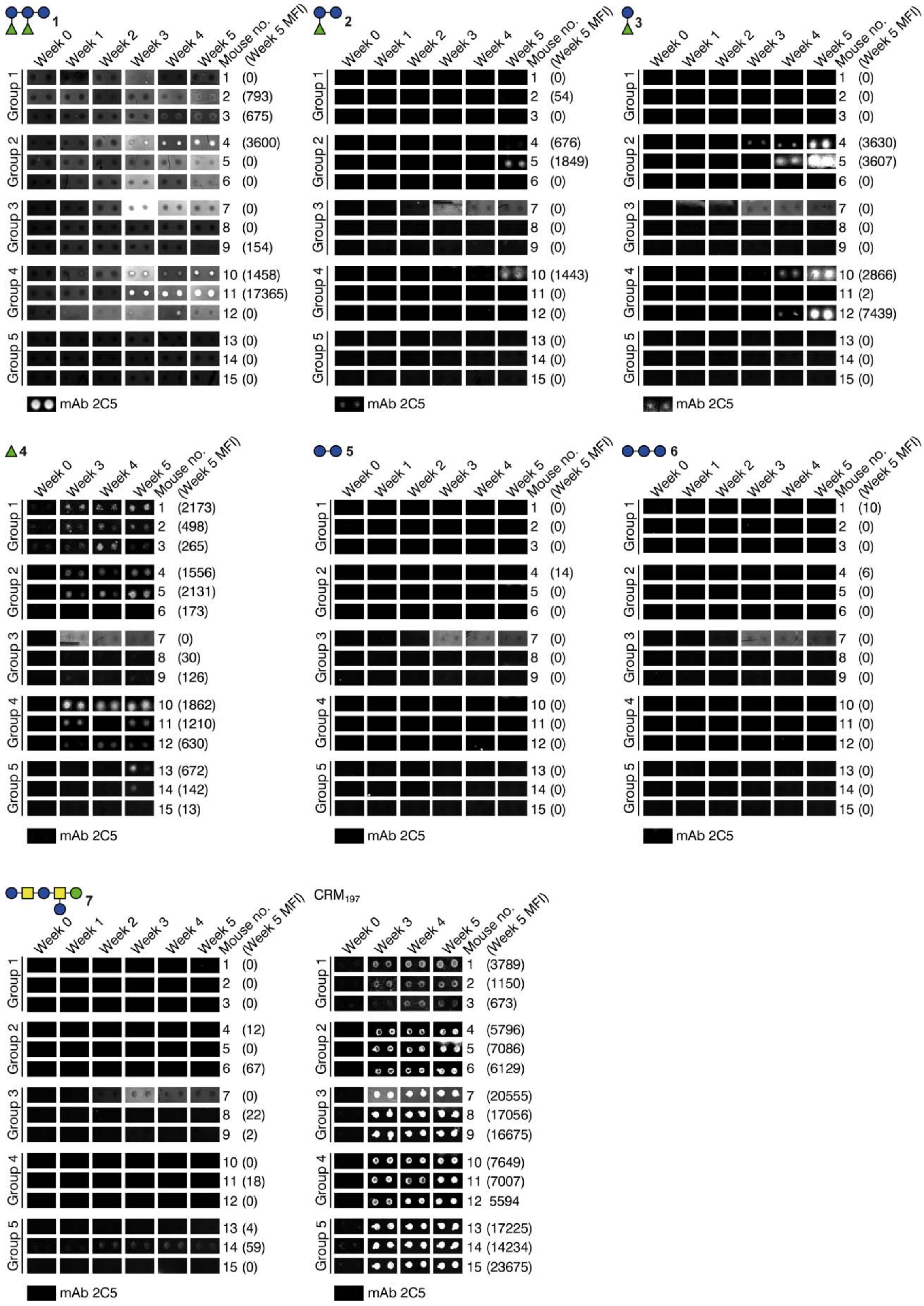

Supplementary Figure 13 | Microarray analysis of mice immunized with 11, 12, and/or 13. Each group comprised three mice. Serum was analyzed at 1:200 dilutions except for pentasaccharide **1**, which was analyzed at 1:20. Background-subtracted MFI signals at week 5 are indicated in parentheses right to the respective scans.

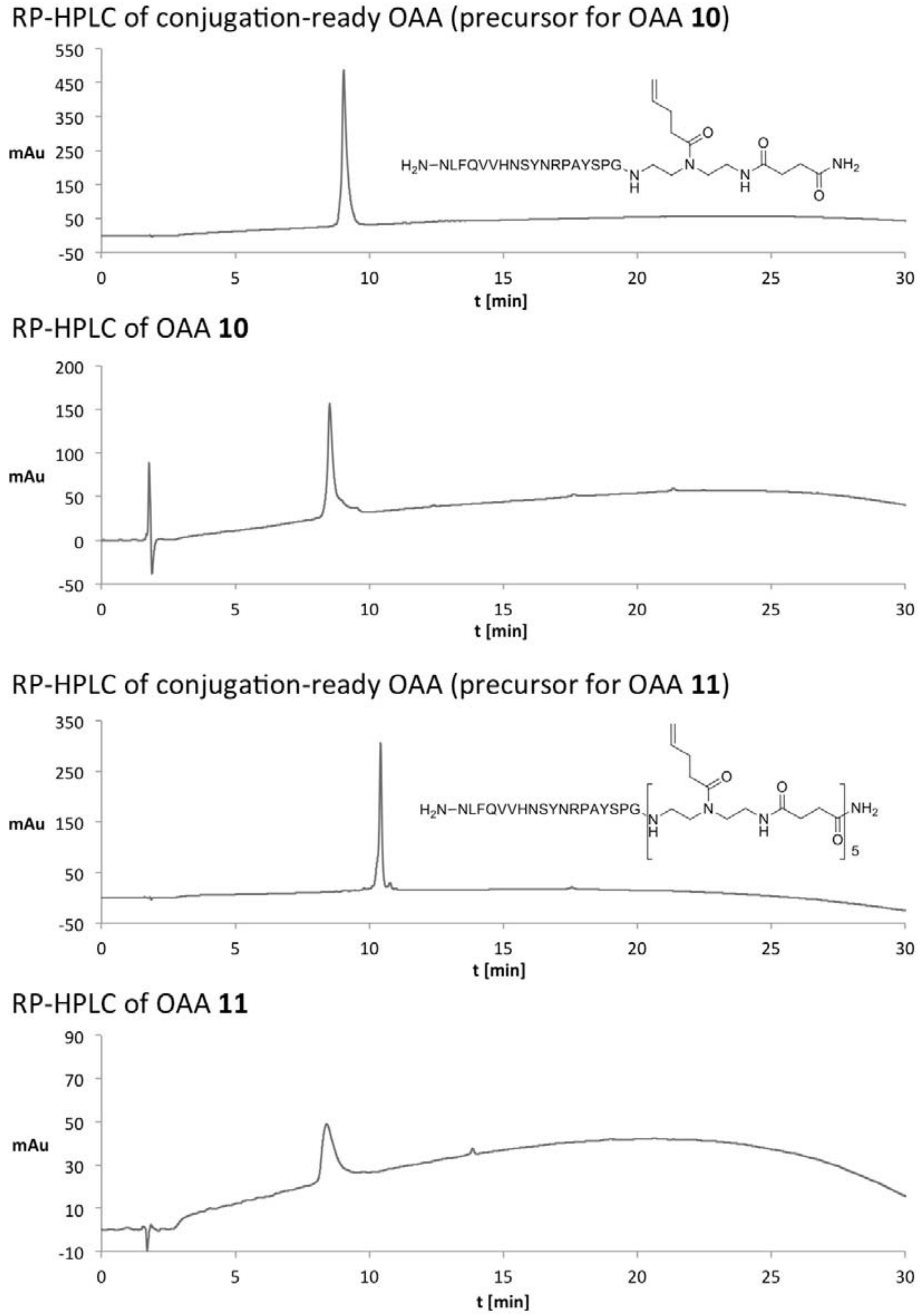

Supplementary Figure 14. | RP-HPLC analysis of OAAs. Conditions: linear gradient 5%-95% B in 30 min; Buffer A = H_2O + 0.1% TFA; Buffer B = MeCN + 0.1% TFA; C18 Agilent Eclipse, 4.6 × 100 mm of conjugation-ready OAAs (precursor for OAA **10** and OAA **11**) and purified OAA conjugate **10** and **11** employed in this study. The sulfoxide pattern of OAA conjugate **11** results in a broader product peak. UV was detected at 214 nm.

MALDI-TOF-MS spectra of conjugation-ready OAA (precursor for OAA **10**)

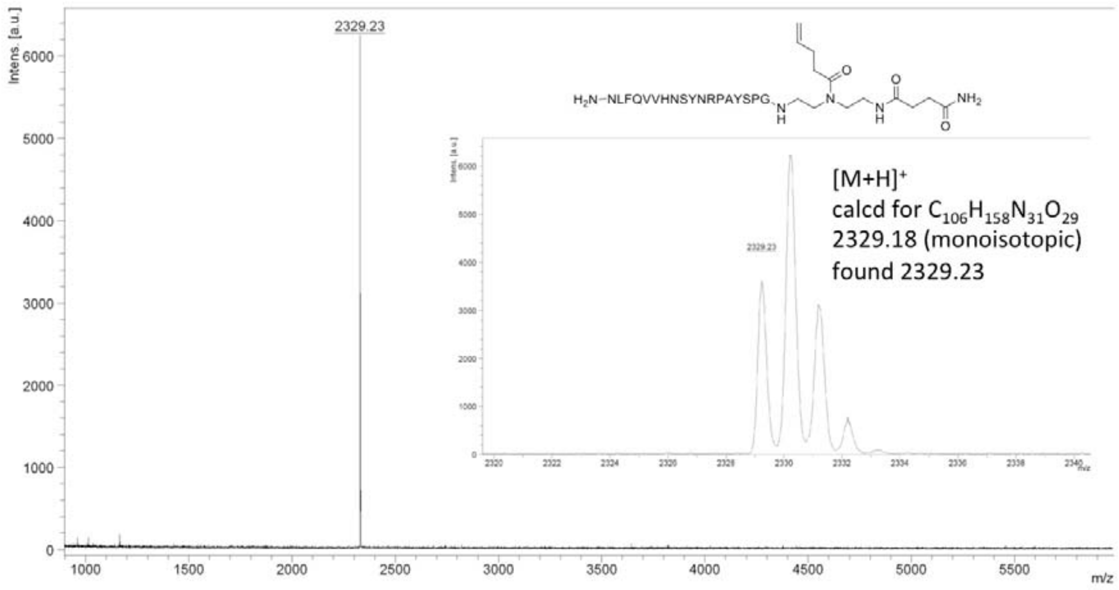

MALDI-TOF-MS spectra of conjugation-ready OAA (precursor for OAA **11**)

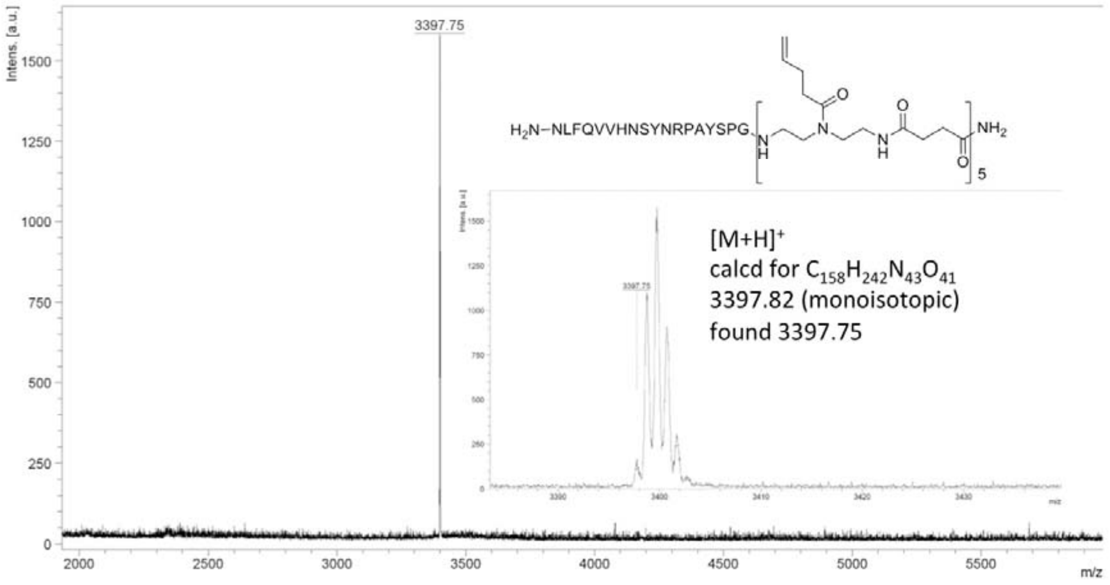

Supplementary Figure 15. | MALDI-TOF-MS spectra of conjugation-ready OAAs (precursor for OAA 10 and 11). MALDI-TOF-MS spectra of OAA conjugate **10** and **11** can be found in Supplementary Fig. 9.

Supplementary Table 1 | Distances between protons for which inter-residue NOEs were observed.

Residue	Proton		Residue	Proton	r (Å)*
Rha D'	H1	<->	Glc C	H3	2.5
Rha D'	H5	<->	Glc C	H3	3.7
Rha D‘	H1	<->	Glc C	H2	4.5
Rha D	H3	<->	Glc C	H6a	2.2
Rha D	H1	<->	Glc B	H2	3.7
Rha D	H3	<->	Glc C	H1	4.6
Glc C	H2	<->	Rha D	H5	2.1
Glc C	H2	<->	Rha D	H6b	2.9
Glc C	H3	<->	Rha D'	H2	4.4
Glc B	H4	<->	Glc C	H1	2.5
Glc B	H3	<->	Rha D	H1	2.7
Glc B	H1	<->	Glc A	H2	2.6
Glc B	H5	<->	Rha D	H1	5.2
Glc B	H4	<->	Rha D	H1	4.6
Glc B	H5	<->	Rha D	H5	4.6
Glc B	H3	<->	Rha D	H5	3.2
Glc B	H6a	<->	Glc C	H1	3.0
Glc B	H6b	<->	Glc C	H1	2.6
Glc B	H5	<->	Glc C	H1	4.1
Glc B	H3	<->	Glc C	H1	4.6
Glc B	H2	<->	Glc C	H1	5.0
Glc B	H1	<->	Glc A	H1	2.6
Glc B	H1	<->	Glc A	H3	4.6

*Distances were derived from the structural model (see Fig. 7d in the main manuscript) and are in agreement with the 5 Å limit of the NOE.

Supplementary Note 1

Synthesis of 2-Thio-ethanol α-l-rhamnopyranosyl-(1→3)-β-D-glucopyranoside **9** (Supplementary Fig. 8) was achieved with the following conditions:

2-(Benzylthio)ethanol 2,3-di-*O*-benzoyl-4-*O*-benzyl-α-L-rhamnopyranosyl-(1→3)-2-*O*-benzoyl-4,6-di-*O*-benzyl-1-thio-β-D-glucopyranoside (S3). Thioglycoside **S1** (ref. 2) (269 mg, 0.25 mmol) and 2-(benzylthio)ethanol **S2** (117 mg, 0,70 mmol) were coevaporated with toluene and kept under high vacuum. The mixture was dissolved in dry DCM (3 mL) and 2,4,6-tri-tert-butylpyridine (TTBPy) (56 mg, 0.23 mmol) and activated 3Å molecular sieves were added. The solution was cooled to 0 °C, treated with DMTST (ref. 3) (130 mg, 0.50 mmol) and stirred for 30 min at 0 °C. The reaction was then warmed to room temperature, diluted with DCM and quenched by addition of sat. aq. $Na_2S_2O_3$ and sat. aq. $NaHCO_3$. The aqueous fraction was extracted with DCM and the combined organic fractions dried over $MgSO_4$ and concentrated. Column chromatography on silica gel (hexanes/EtOAc) gave **S3** (266 mg, 0.16 mmol, 65%). $[\alpha]_D^{20}$ = + 62.5 º (c = 3.8, $CHCl_3$), IR ν_{max} (film) 2871, 1728, 1601, 1451, 1264, 1094, 1069, 1027 cm^{-1}; ^{1}H-NMR (400 MHz, $CDCl_3$) δ 8.06 – 7.94 (m, 2H), 7.90 – 7.78 (m, 2H), 7.74 – 7.65 (m, 2H), 7.49 (ddd, *J* = 22.4, 10.6, 4.3 Hz, 2H), 7.36 – 7.15 (m, 27H), 5.72 (dd, *J* = 9.4, 3.4 Hz, 1H), 5.45 (dd, *J* = 3.4, 1.9 Hz, 1H), 5.37 (dd, *J* = 9.1, 7.9 Hz, 1H), 5.23 (d, *J* = 1.8 Hz, 1H), 4.87 (d, *J* = 10.6 Hz, 1H), 4.70 – 4.58 (m, 2H), 4.54 – 4.50 (m, 2H), 4.19 – 4.12 (m, 2H), 3.94 (dt, *J* = 10.2, 6.7 Hz, 1H), 3.85 – 3.80 (m, 2H), 3.68 – 3.63 (m, 2H), 3.59 (d, *J* = 2.7 Hz, 2H), 2.63 (t, *J* = 6.0 Hz, 3H), 2.53 (t, *J* = 6.9 Hz, 2H), 2.09 (t, *J* = 6.1 Hz, 2H), 1.11 (d, *J* = 6.2 Hz, 3H); ^{13}C-NMR (100 MHz, $CDCl_3$) δ 165.28, 164.97, 164.75, 138.50, 138.09, 137.97, 137.53, 133.12, 133.07, 132.89, 130.00, 129.93, 129.82, 129.77, 129.73, 129.68, 129.02, 128.58, 128.52, 128.44, 128.40, 128.37, 128.32, 128.30, 128.21, 128.00, 127.98, 127.84, 127.80, 127.75, 127.00, 100.97, 97.61, 79.23, 77.81, 77.36, 76.79, 75.65, 75.60, 74.97, 74.34, 73.77, 71.93, 71.24, 69.26, 68.75, 68.33, 36.60, 30.57, 18.00; HRMS (ESI): Calcd for $C_{63}H_{62}O_{13}SNa^+$ $[M+Na]^+$ 1081.3803, found 1081.3800.

2-Thio-ethanol α-L-rhamnopyranosyl-(1→3)-β-D-glucopyranoside (9). To a solution of **S3** (82 mg, 77 µmol) in THF (2 mL) and MeOH (0.5 mL) was added NaOMe (0.5 ml, 0.5M in MeOH) and the mixture was stirred for 1 h. The mixture was neutralized with Amberlite IR 120 (H^+) ion exchange resin, filtered and concentrated. The triol product was purified by size exclusion chromatography (Sephadex LH-20, $CHCl_3$/MeOH 1:1) and concentrated. To a solution of liquid ammonia (40 mL) was added at -78 °C a solution of the triol in THF (7 mL). The mixture was treated with tBuOH (0.13 mL) and sodium (65 mg) was added portion wise until a deeply blue color persisted. The reaction was stirred at -78 °C for 35 min and quenched by addition of MeOH, followed by solid NH_4OAc. The solution was warmed to room temperature under a stream of nitrogen and concentrated. Size exclusion chromatography (Sephadex LH-20, MeOH) gave **9** (24 mg, 62 µmol, 81%). ^{1}H-NMR (400 MHz, D_2O) δ 5.15 (d, J = 1.3 Hz, 1H), 4.52 (d, J = 8.1 Hz, 1H), 4.10 – 3.98 (m, 3H), 3.96 – 3.90 (m, 1H), 3.88 – 3.78 (m, 2H), 3.77 – 3.70 (m, 1H), 3.61 (t, J = 9.0 Hz, 1H), 3.53 – 3.37 (m, 4H), 2.78 (t, J = 6.3 Hz, 2H), 1.26 (d, J = 6.3 Hz, 3H); ^{13}C-NMR (100 MHz, D_2O) δ 104.46, 103.52, 84.59, 78.39, 76.19, 74.43, 74.18, 72.78, 72.65, 71.29, 70.53, 63.19, 25.68, 18.95; HRMS (ESI): Calcd for $C_{14}H_{26}O_{10}SNa^+$ $[M+Na]^+$ 409.1139, found 409.1137.

S3: ^{1}H NMR ($CDCl_3$, 400 MHz)

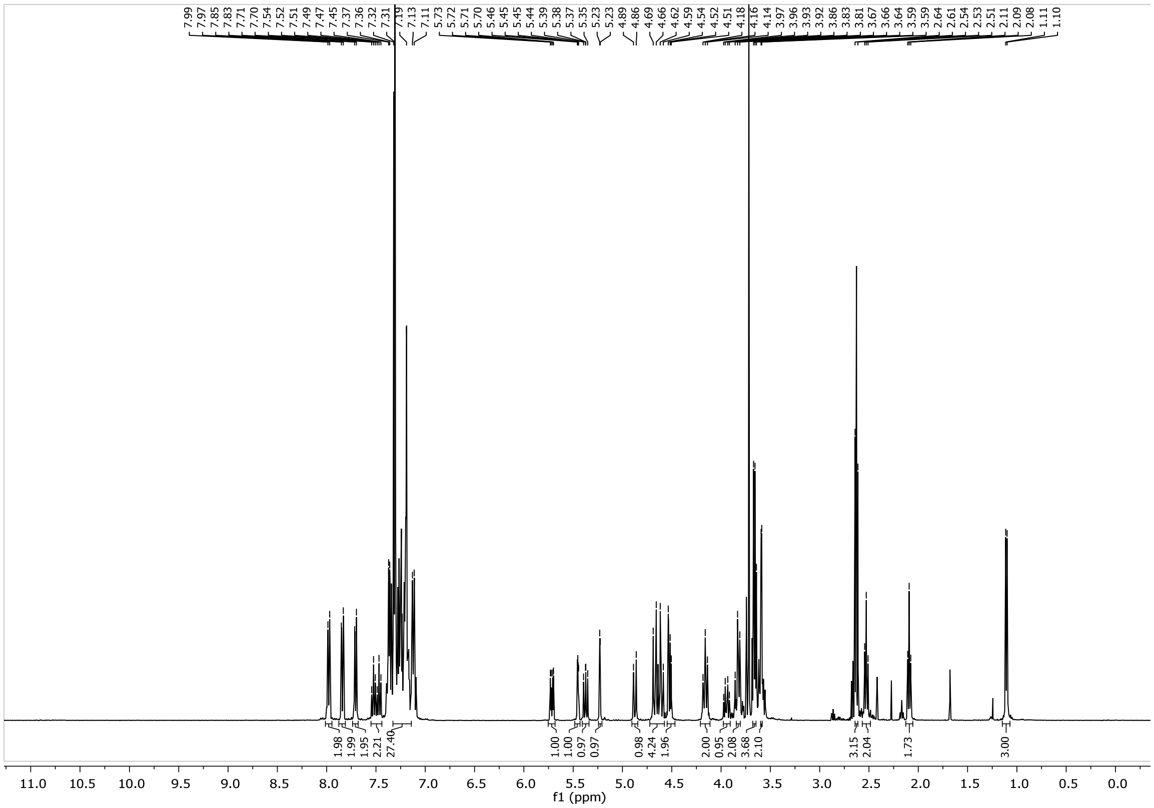

S3: ^{13}C NMR ($CDCl_3$, 400 MHz)

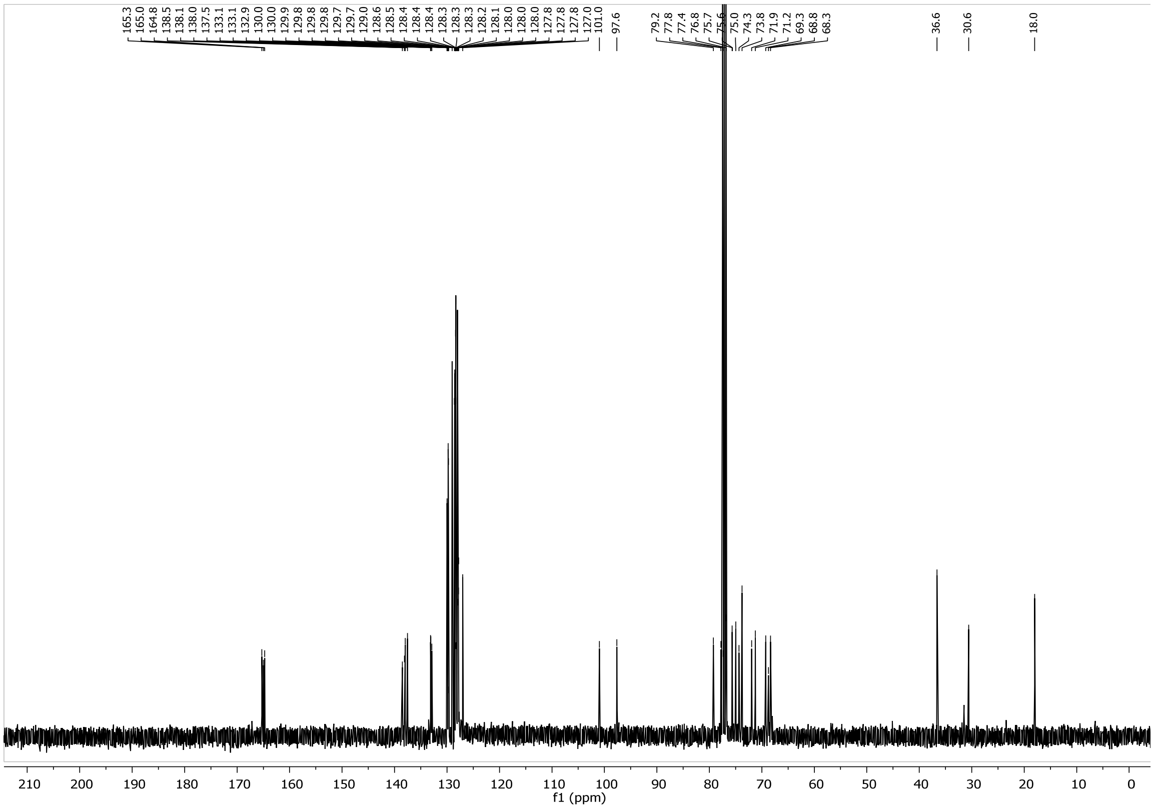

9: ^{1}H NMR (D_2O, 400 MHz)

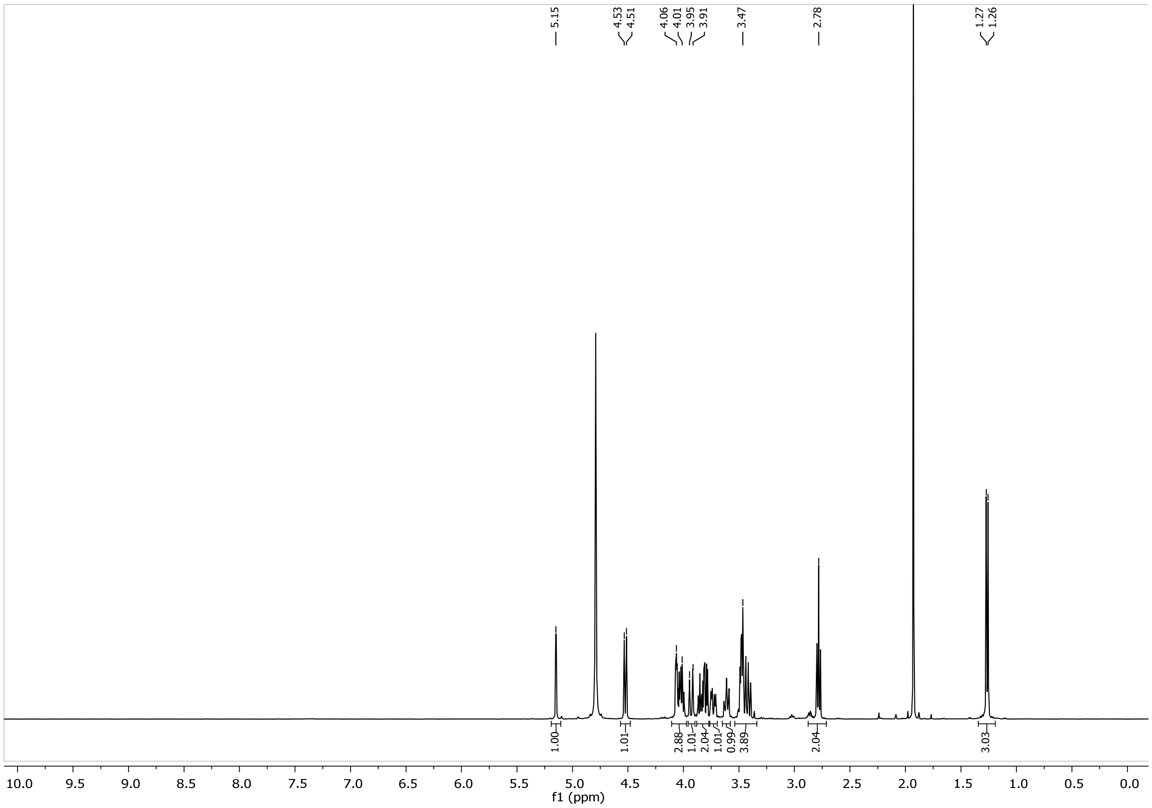

9: ^{13}C NMR (D_2O, 400 MHz)

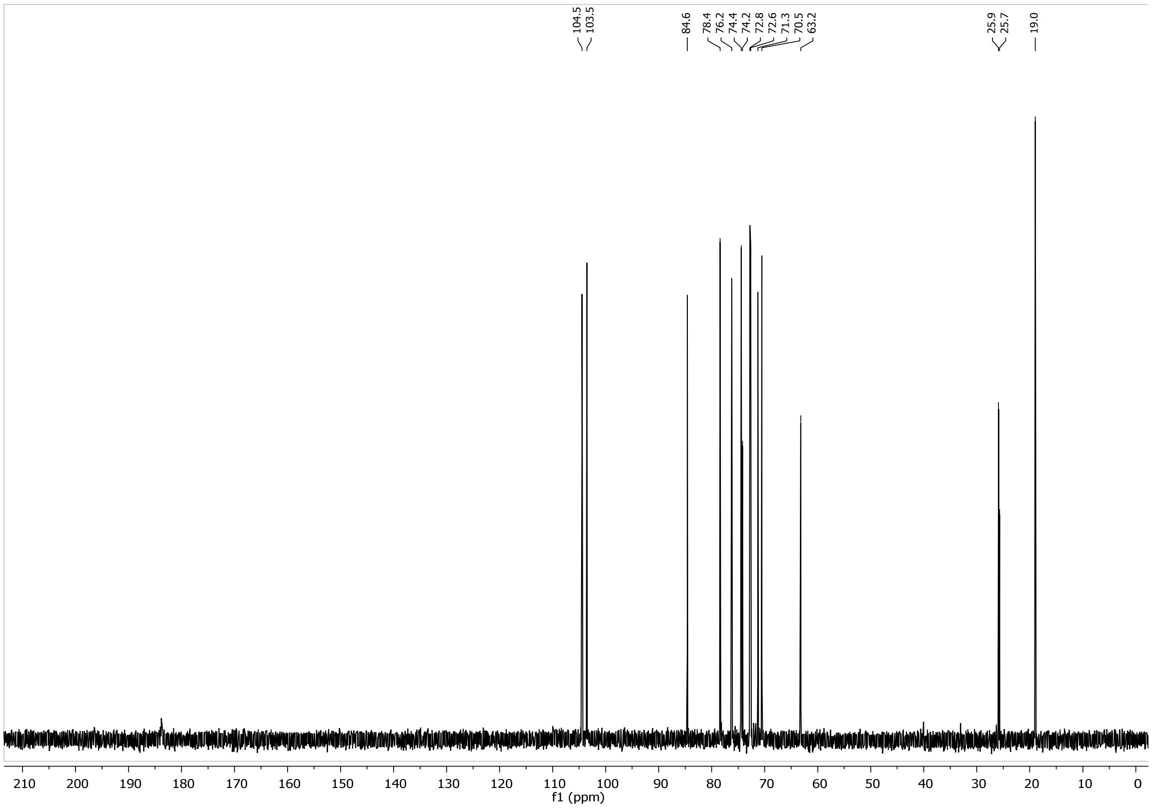

S3: HRMS (ESI)

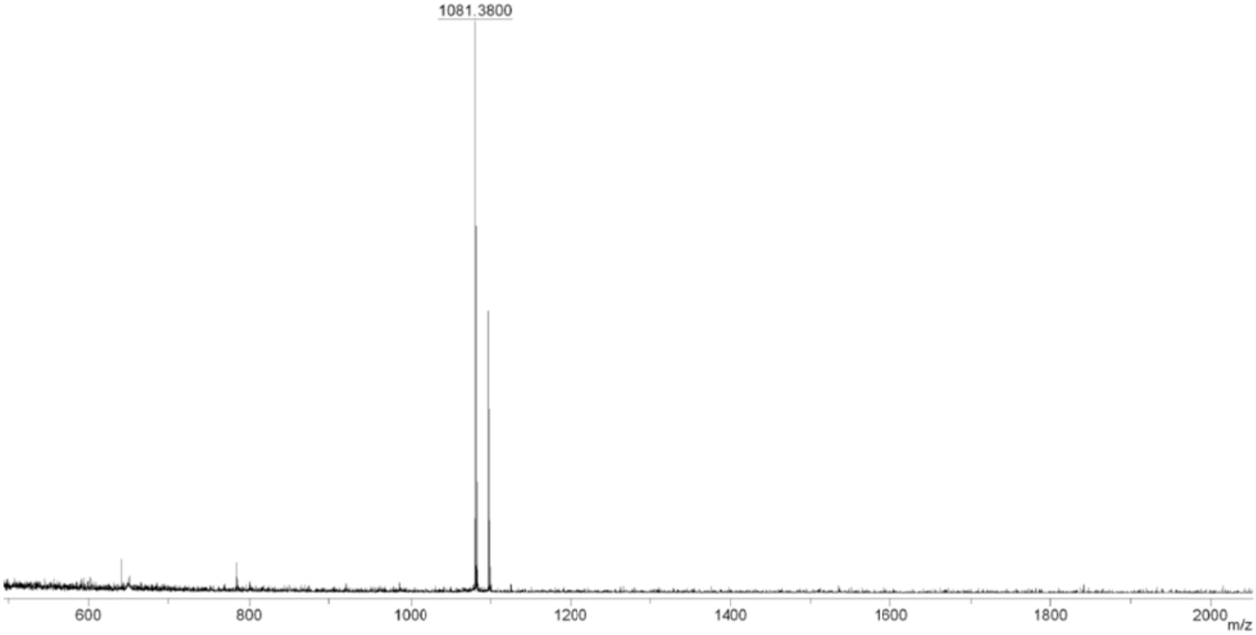

9: HRMS (ESI)

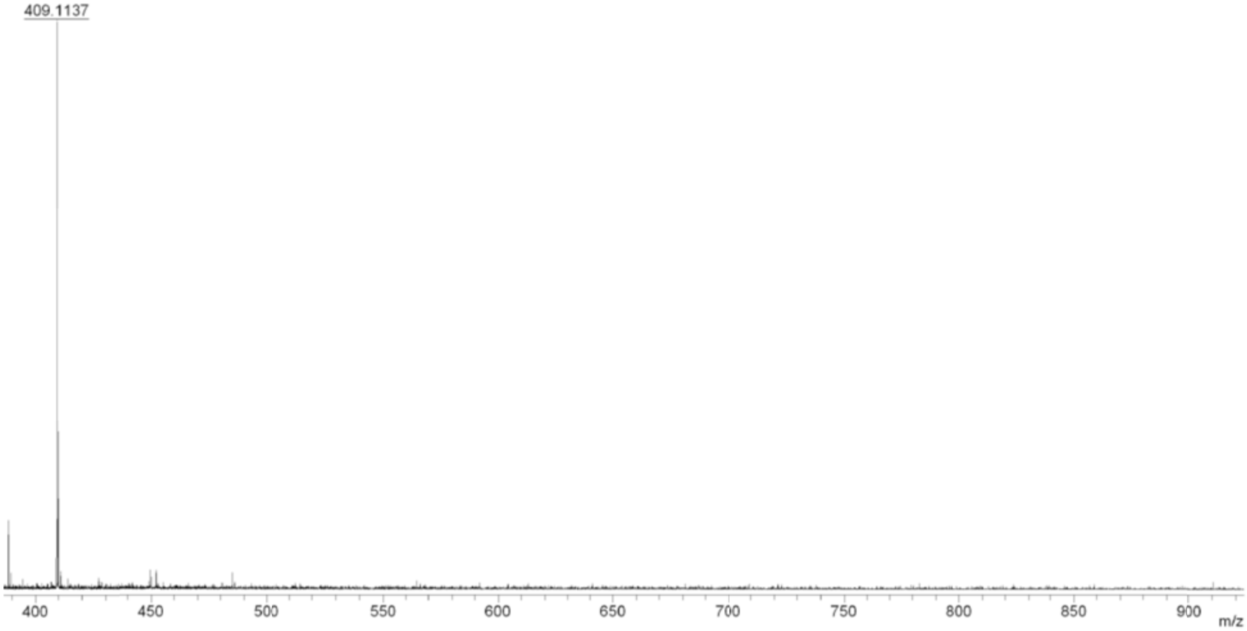

Supplementary Note 2

Synthesis and characterization of glycoconjugates 12 and 13. Glycoconjugates **12** and **13** were synthesized with a protocol modified from Wu *et al.*[1] by using di-*p*-Nitrophenyl adipate ester as spacer molecule. This spacer was chosen over the previously used di-*N*-Succinimidyl adipate ester[2] to facilitate the challenging conjugation reaction with construct **11**. The reaction schemes are shown in Supplementary Fig. 10a for **12** composed of disaccharide **3** and CRM_{197} and Supplementary Fig. 10b for glycoconjugate **13** composed of construct **11** and CRM_{197}.

The resulting glycoconjugates were analyzed by SDS-PAGE (Supplementary Fig. 10c). Shifts towards higher masses of **12** and **13** compared to non-conjugated carrier protein CRM_{197} indicated successful conjugation reactions. To detect the glycan antigens on the glycoconjugates, western blots (WB) were performed (Supplementary Fig. 10d). Ponceau S staining confirmed successful transfer to the WB membrane and verified the mass shifts observed by SDS-PAGE. Anti-diphtheria toxin antibody bound to both **12** and **13** as well as CRM_{197}, as expected since diphtheria toxin and CRM_{197} only differ by one amino acid substitution. In contrast, anti-PS-I antibodies (an equimolar combination of mAbs 2C5, 10A1 and 10D6) stained the glycoconjugates only. This verified that PS-I glycans were present on **12** and **13**. The signal intensity to **12** was much stronger than to **13**, leading to saturation signals for **12**. This was due to the comparably high glycan loading of **12** indicated by increased mass shifts observed in SDS-PAGE and WB. Single disaccharides **3** may also be more accessible to the anti-PSI mAbs than disaccharides part of pentavalent constructs in **13**. To estimate the carbohydrate loading of **12** and **13**, colorimetric anthrone assays were performed (Supplementary Fig. 10e) with a modified procedure described before for glycan analysis of CRM_{197} glycoconjugates[4]. As expected, the CRM_{197} carrier protein had no measurable carbohydrate content. In contrast, glycoconjugates **12** and **13** showed an average of 140.6 μg and 46.3 μg total carbohydrate content per mg protein, respectively. This further verified successful conjugation reactions. Based on the anthrone assays, conjugation ratios were calculated to 20 disaccharides **3** per mol of CRM_{197} (**12**) and 1.3 constructs **11** per mol of CRM_{197} (**13**), the latter corresponding to 6.5 disaccharide units per mol of CRM_{197}.

Immunization studies with of pentavalent construct 11 and glycoconjugates 12 and 13. The two glycoconjugates and construct **11** were used to immunize five groups of mice. Regimes of subcutaneous immunizations in two-week intervals were chosen[2]. Group 1 was immunized three times with **11**, group 2 three times with **12**, group 3 three times with **13**, group 4 twice with **12** and then once with **13**, and group 5 twice with **13** and then once with **12** (Supplementary Fig. 11a). Groups 4 and 5 were included to detect cross-reactions between immunogens **12** and **13**.

Serum IgGs in the immunized mice were detected by glycan microarray experiments (Supplementary Figs 11b and 13). A subset of mice of groups 1, 2 and 4 produced IgGs to pentasaccharide **1**. In group 3, only a very weak IgG response to **1** was observed in one mouse after the third immunization, whereas anti-**1** IgGs were undetectable in group 5. IgG responses to disaccharide **3** and trisaccharide **2** were seen in groups 2 and 3 only. This confirmed our previous results that a **3**-CRM_{197} glycoconjugate was capable of eliciting IgGs cross-reacting with larger PS-I glycans[2]. In contrast to the glycoconjugate **12**, construct **11** elicited IgGs to pentasaccharide **1** only and not to smaller glycans **2** and **3**. Glycoconjugate **13** proved to be unable to generate IgGs to PS-I glycans **1**-**3** by itself (groups 3 and 5), but we observed a boosting of IgG responses to glycans **1**-**3** elicited by two prior immunizations with **12** (group 4). IgGs to mono-rhamnose **4** were observed in all groups, which confirmed our previous studies where antibodies to **4** were elicited with **3**-CRM_{197} glycoconjugate[2]. Anti-**4** IgG responses were substantially weaker in groups 3 and 5, further supporting the notion that **13** was unable to efficiently generate glycan-specific antibodies. IgGs to oligoglucoses **5** and **6** were undetectable in all groups, supporting our findings that the glucose backbone of PS-I *per se* is immunologically inert (ref. 2 and this study). Also, no cross-reaction to the structurally non-related PS-II glycan of *C. difficile* was observed, indicating the high specificity of glycan-specific IgGs to PS-I glycans. IgGs to the CRM_{197} carrier protein were observed in all mice, demonstrating successful immunization and the functionality of the synthetic T-cell epitope of **11**.

IgG responses to **1** and **3** were confirmed by SPR experiments. The glycans were immobilized on SPR sensor chips and pooled serum dilutions at week 5 post-immunization were passed through the flow cells to detect glycan-specific antibodies. Non-specific binding of serum components was accounted for by subtracting response unit signals of pooled serum at week 0 of the respective groups to BSA-functionalized flow cells. Similar to the observations seen in glycan microarray experiments described above, antibodies to **1** were seen in groups 1, 2 and 4 (Supplementary Fig. 12a), and those to **3** only in groups 2 and 4 (Supplementary Fig. 12b). Again, antibodies to both glycans were undetectable in groups 3 and 5.

Supplementary References

[1] Wu, X., Ling, C. C., Bundle, D. R. A new homobifunctional p-nitro phenyl ester coupling reagent for the preparation of neoglycoproteins. *Org. Lett.* **6**, 4407-4410 (2004).

[2] Martin, C. E. *et al.* Immunological evaluation of a synthetic *Clostridium difficile* oligosaccharide conjugate vaccine candidate and identification of a minimal epitope. *J. Am. Chem. Soc.* **135**, 9713-9722 (2013).

[3] Schumann, B. *et al.* Synthesis of conjugation-ready zwitterionic oligosaccharides by chemoselective thioglycoside activation. *Chem. Sci.* **5**, 1992-2002 (2014).

[4] Turula, V. E. Jr., Gore, T., Singh S, Arumugham, R. G. Automation of the anthrone assay for carbohydrate concentration determinations. *Anal. Chem.* **82**, 1786-1792 (2010).
